# Comparative assessment of methods for the computational inference of transcript isoform abundance from RNA-seq data

**DOI:** 10.1186/s13059-015-0702-5

**Published:** 2015-07-23

**Authors:** Alexander Kanitz, Foivos Gypas, Andreas J. Gruber, Andreas R. Gruber, Georges Martin, Mihaela Zavolan

**Affiliations:** Biozentrum, University of Basel and Swiss Institute of Bioinformatics, Basel, Switzerland

## Abstract

**Background:**

Understanding the regulation of gene expression, including transcription start site usage, alternative splicing, and polyadenylation, requires accurate quantification of expression levels down to the level of individual transcript isoforms. To comparatively evaluate the accuracy of the many methods that have been proposed for estimating transcript isoform abundance from RNA sequencing data, we have used both synthetic data as well as an independent experimental method for quantifying the abundance of transcript ends at the genome-wide level.

**Results:**

We found that many tools have good accuracy and yield better estimates of gene-level expression compared to commonly used count-based approaches, but they vary widely in memory and runtime requirements. Nucleotide composition and intron/exon structure have comparatively little influence on the accuracy of expression estimates, which correlates most strongly with transcript/gene expression levels. To facilitate the reproduction and further extension of our study, we provide datasets, source code, and an online analysis tool on a companion website, where developers can upload expression estimates obtained with their own tool to compare them to those inferred by the methods assessed here.

**Conclusions:**

As many methods for quantifying isoform abundance with comparable accuracy are available, a user’s choice will likely be determined by factors such as the memory and runtime requirements, as well as the availability of methods for downstream analyses. Sequencing-based methods to quantify the abundance of specific transcript regions could complement validation schemes based on synthetic data and quantitative PCR in future or ongoing assessments of RNA-seq analysis methods.

**Electronic supplementary material:**

The online version of this article (doi:10.1186/s13059-015-0702-5) contains supplementary material, which is available to authorized users.

## Background

The general availability of high-throughput sequencing technologies greatly facilitated the detection and quantification of RNA species, including protein-coding RNAs, long non-coding RNAs, and microRNAs, in many different systems. In higher eukaryotes, the vast majority of protein-coding genes express multiple transcript isoforms [1–3]. Although a substantial proportion of transcript isoforms may result from stochasticity in the splicing process [4, 5], striking examples of isoform switching with large impact on cellular phenotypes are also known (for example, [6, 7]). Tissue-specific splicing patterns have been linked to the expression of specific RNA-binding proteins [8], some of which appear to act as ‘master’ regulators of alternative splicing in individual tissues [9]. For example, muscleblind-like proteins 1 and 2 (MBNL1/MBNL2) are expressed in mesenchymal cells and their downregulation facilitates somatic cell reprogramming [10], while the epithelial splicing regulatory proteins 1 and 2 (ESRP1/ESRP2) establish epithelia-specific patterns of isoform expression [11]. Nevertheless, despite the long history of the field, the functional relevance of most isoforms that can be detected with sequencing approaches remains unclear [12], particularly in light of the rapid change of isoform usage pattern in evolution that indicates relatively weak selection pressure [13].

Analysis of expression pattern is often one of the first steps towards understanding a gene’s function. However, transcript isoform abundance is almost always quantified indirectly; most of the sequencing technologies that are currently used yield reads that are short (≤200 nt) relative to the length of eukaryotic transcripts (2.2 kb in mammals, on average) [14] and thus, a sequenced read can typically be assigned to more than one isoform. This is not the case with the technology developed by Pacific Biosciences that enables sequencing of full-length cDNAs [15]. A drawback of this technology is, however, that the throughput is relatively low, of the order of 10^4^ transcripts, which does not allow accurate quantification of transcript abundance. Furthermore, the error rates are relatively high, making the transcript identification non-trivial. Thus, accurate and cost-effective quantification of the complete repertoire of full-length expressed transcripts, which are in the range of hundreds of thousands per cell [16], remains an open problem.

As RNA sequencing (RNA-seq) has become commonplace in molecular biology laboratories, a variety of computational approaches has been proposed for isoform reconstruction from short read sequencing data (see, for example, [17]). Similarly, quite a number of methods has been developed for the inference of isoform abundance (reviewed in [18]). While short read alignment and transcript reconstruction methods have been extensively benchmarked recently [17, 19, 20], only one study, rather limited in scope, evaluated some isoform quantification methods [21]. Independently and comprehensively evaluating the accuracy of such computational methods is difficult, because experimental validation strategies by, for example, quantitative PCR are typically restricted to just a limited number of isoforms (see, for example, [22]). Developers therefore typically evaluate their tools on synthetically generated datasets which may not capture adequately the complexities of RNA-seq experiments.

In this study we carried out a systematic evaluation of a large number of methods for isoform quantification from RNA-seq data. We used not only synthetic, but also genome-wide experimental datasets. We took advantage of newly developed protocols for quantifying the abundance of distinct RNA 3′ ends, which result from the use of alternative 3′ end processing sites. These protocols allow a comprehensive surveillance of 3′ end processing site usage, with a method that is distinct from RNA-seq [23–25]. From two types of cells and from two species (human Jurkat T cells or mouse NIH/3T3 cells) we prepared two libraries, one with an RNA-seq protocol and the other with a protocol for capturing the 3′ ends of polyadenylated RNAs. We submitted the aligned RNA-seq reads to the entire panel of computational methods for estimation of transcript isoform abundance. We then compared these estimates with those that we obtained independently, through the analysis of the corresponding 3′ end sequencing data.

Our results indicate that many of the available methods have comparable accuracy, and that the abundance of highly expressed isoforms is more accurately inferred than the abundance of isoforms with low expression levels. We further found that even the quantification of gene expression is more accurate when gene expression levels are computed by cumulating the levels of transcript isoforms than when ignoring the transcript structures. Given that many methods are available that differ little in accuracy, a user’s choice will likely be determined by factors such as the memory and runtime requirements, as well as the availability of methods for downstream analyses such as differential gene/transcript expression.

## Results

We initially performed an extensive literature survey to identify tools that were developed for inferring the abundance of transcript isoforms from RNA-seq data. Although we tried to include as many of these as possible, our study setup required that tools are able to quantify a set of transcripts that we provided as input, thereby separating the problem of transcript reconstruction from that of abundance quantification. To be able to interpret the results, we further focused on methods that have been duly described in the literature. Lastly, we thought that ease of use would be critical for the adoption of the tool by the user community and we did not pursue methods which we were unable to implement within a reasonable amount of time. Table 1 lists the remaining 11 tools, together with their underlying principle, input requirements, and references. A description of how each of the tools was applied is provided in the Methods section.

**Table 1:** Overview of surveyed methods

Name	Reference sequence^a^	Principle	Released
BitSeq	Transcripts	Bayesian estimation of parameters of a model that explains the read-to-transcript alignment data. Reads are assumed to be sampled independently, without positional bias from transcripts, such that the probability of an alignment starting at a given position of a transcript is inversely proportional to the transcript length. Sub-optimal alignments are used to estimate the ‘background’ of spurious alignments.	2012 [67, 68]
CEM	Genome	Component elimination expectation-maximization approach to estimating the parameters of isoform abundance. For each gene it aims to find a ‘sparse’ solution, with few expressed isoforms. Read sampling from isoforms is assumed to obey a quasi-multinomial distribution, in which positional and other biases are modeled as an effective distribution which could be, for example, uniform (no positional bias) or exponential (modeling the process of RNA degradation).	2012 [69]
Cufflinks	Genome	Bayesian approach to estimating transcript abundances by explicitly modeling the length of the fragments expected from RNA-seq. It assumes that for a given gene, reads are sampled independently with uniform probability along transcripts and in proportion to the transcript abundance between transcripts. Thus, if a read can be assigned to two transcripts of different lengths, the transcript with a shorter effective length will have a higher probability of giving rise to the read.	2010 [70]
eXpress	Transcripts	Similar to Cufflinks, but it includes modeling of errors and indels and it has a different model for fragment length selection. Unlike Cufflinks and most other methods, eXpress processes read alignments ‘on-line’ so that it can be integrated into real-time analysis pipelines.	2012 [32]
IsoEM	Genome	Expectation-maximization approach to inferring isoform abundances that are consistent with the coverage of isoforms by reads. The coverage is assumed to be uniform along an isoform. Base quality scores are taken into account in computing the probabilities of alignments. In the E-step, the expected number of reads derived from a given isoform is computed and in the M-step, the relative frequencies of isoforms are estimated.	2011 [71]
MMSeq	Transcripts	Models the read data as Poisson-distributed variables with rates that depend on the abundance of the regions of the transcripts with which the reads are compatible and on the sequence-dependent bias in capturing the sequences. Priors on transcript abundances are Gamma-distributed. Sequencing errors are not modeled, there is only a filter on the minimal quality of considered alignments.	2011 [73]
RSEM	Transcripts	Models the probability of observing a read as the sum of the relative abundance of the transcript to which the reads maps times the probability of the read mapping to the transcript, and infers transcript abundances by expectation maximization.	2009 [34, 35]
rSeq	Transcripts	Models read data as Poisson-distributed variables with rates that depend on the abundance of the regions of the transcripts with which the reads are compatible.	2009 [75]
Sailfish^b^	Transcripts	Expectation-maximization method for explaining the abundance of k-mers inferred from the reads in terms on the abundance of the transcripts with the associated k-mer abundances.	2014 [76]
Scripture	Genome	Transcript abundance is calculated as reads per kilobase of exonic sequence per million aligned reads, given the alignments of the reads to the genome and the annotated/reconstructed transcript.	2010 [77]
TIGAR2	Transcripts	Models the read data in terms of a large number of parameters which include, beyond the relative abundance of the transcripts, the read length distribution, the nucleotides, and alignment state and quality at the first and second position of the read.	2013 [78, 85]

The columns are: method name, sequences to which reads are compared (transcripts or genome), principle of the method, year of release, and associated reference(s)

^a^For methods operating on the genome sequence, genome annotation files (GTF/BED-formatted) were also provided

^b^In contrast to other methods operating on transcripts, Sailfish uses k-mer statistics rather than aligning reads to transcripts

### Runtime and memory requirements differ substantially between tools

**Fig. 1:**
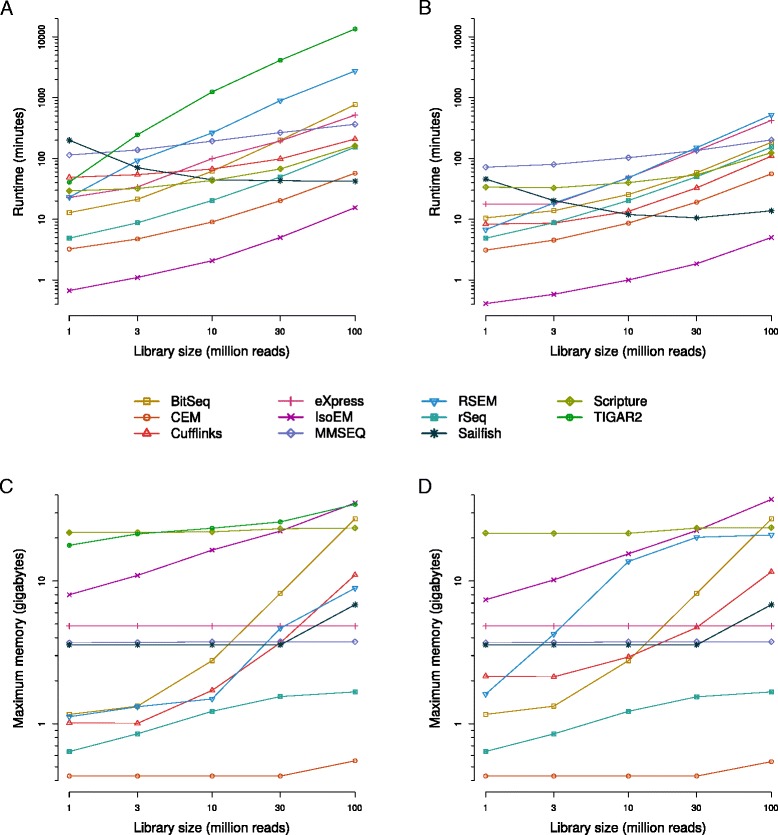
Running time and memory requirements. Transcript isoform abundances were estimated with each of the indicated methods from *in silico*-generated datasets of different ‘sequencing’ depths. The running times (**a** and **b**) and memory footprints (**c** and **d**) are shown as a function of sequencing depth. Programs were run on either one (**a** and **c**) or 16 cores (**b** and **d**). Note that TIGAR2 is missing in (**b**) and (**d**), because the method does not support the use of multiple cores

Most of the tools that we surveyed have previously been tested by the developers on simulated data. Here, we have used the Flux Simulator software [26] to generate reads corresponding to GENCODE-annotated transcripts (Additional file 1: Figure S1). To assess how the runtime complexity, memory requirements, and accuracy of the different programs depended on the sequencing depth we generated sets of 1, 3, 10, 30, and 100 million single-end reads, the latter two values being in the range that is currently obtained from sequencing a typical RNA-seq library on broadly used next-generation sequencing platforms. We found that the tested programs differ substantially in their runtimes and memory footprints, as measured under defined conditions on a dedicated machine (maximum available memory = 64 Gb). As shown in Fig. 1, the CPU times necessary to process the different datasets span about three orders of magnitude when a single processor is used (Fig. 1a), and two orders of magnitude when the multi-threading option (16 cores; Fig. 1b) is used. In particular, the times required to process the alignments of 100 million *in silico*-generated reads range between approximately 7 min (IsoEM) and more than 1 week (TIGAR2) when a single processor is used, and between about 5 min (IsoEM) and 8 h (RSEM) when 16 cores are available for the tools that support multi-threading (TIGAR2 does not). With the exception of Sailfish, runtimes strictly increased with the number of processed read alignments. Assuming that a method-specific, but largely sample size-independent time span is required to index the supplied transcriptome, time complexities for most of the quantification algorithms appear to be approximately linear. Sailfish’s runtimes seem to be the highest for the smallest dataset, presumably because the convergence of estimation is slow for small datasets, when the vast majority of transcripts are sparsely covered. Notably, Sailfish computes abundances based on raw read sequences rather than alignments. Thus, whenever alignments are dispensable, a considerable amount of time (typically 1 h or more) can be saved on sample pre-processing compared to all other methods (refer to [19, 27, 28] for an overview of ‘mapping’ times for some short-read aligners and conditions). Enabling multithreading had only a limited impact on runtimes (Additional file 2: Figure S2A), with several of the tools hardly benefiting at all (maximum ratio between runtimes at 1 and 16 cores approximately two-fold or less for CEM, eXpress, MMSEQ, rSeq, and Scripture). However, RSEM (approximately 5.9-fold speedup for 30 million reads) and BitSeq (approximately 4.2-fold speedup for 100 million reads), two methods with the highest single-processor running times had the highest speedup when multiple processors were provided. Memory footprints also spanned almost two orders of magnitude between tools, both when using a single or multiple cores (Fig. 1c, d). For approximately half of the tools (CEM, eXpress, MMSEQ, Sailfish, Scripture, TIGAR2) the memory footprint seems to be largely independent of the sample size. For the remaining tools (BitSeq, Cufflinks, IsoEM, RSEM, rSeq) the memory footprint increases with the sample size. Although IsoEM seems to trade off a relatively large memory footprint (from <10 to >30 GB) for extremely short running times, we did not observe a general inverse correlation between the running time and memory usage of individual methods (r_s_ = 0.13 and −0.13 at 100 million reads for 1 and 16 cores, respectively) (Additional file 2: Figure S2B, C).

### Most methods infer transcript abundances with good accuracy even from sparse datasets

**Fig. 2:**
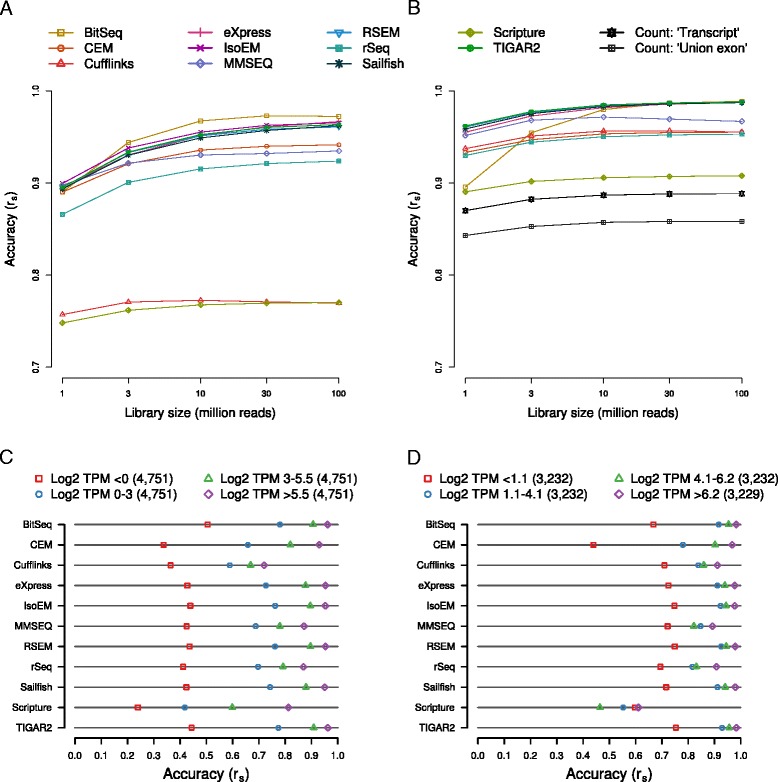
Influence of sequencing depth and expression levels on the accuracy of expression estimates. Transcript isoform and gene expression levels were estimated with each of the indicated methods from *in silico*-generated datasets of different ‘sequencing’ depths. The accuracy of a method was assessed in terms of the Spearman correlation coefficient (r_s_) between the estimates and the known input levels (‘ground truth’) of expressed transcripts (**a**) and genes (**b**). Based on their true abundances, transcripts (**c**) and genes (**d**) were distributed across four bins of expression levels. Estimation accuracies as in (**a**) and (**b**) are indicated for each method and bin. The numbers of transcripts and genes in each bin are indicated together with the expression ranges that they cover. Estimates are based on a sequencing depth of 30 million reads

Our main objective was to evaluate the accuracy of isoform expression estimates produced by various methods. Consistent with current expectations about the number of expressed transcripts in a given cell type, the read simulation software only assigned non-zero expression to approximately 10.2 % of all transcripts supplied to it as input (19,004 out of 187,176). To avoid the situation that our results are dominated by how different methods handle transcripts that are essentially not expressed, we initially restricted our initial analysis to the set of expressed transcripts. These were those for which the simulation software assumed non-zero expression values. When comparing the abundances of these transcripts as inferred by each method with the ‘ground truth’ (Fig. 2a and Additional file 3: Figure S3), we found that nine out of 11 programs exhibit very good performance (Spearman correlation coefficient r_s_ >0.9 for ≥10^7^ reads). As expected, correlations generally improved with increasing library sizes, in a monotonic fashion and asymptotically towards saturation. For most methods, estimation accuracies reached a plateau at or around a read depth of 30 million reads, indicating that further increases in read depth are unlikely to significantly improve their results. In particular, Spearman correlation coefficients peaked at above 0.95 for six of the methods (BitSeq, eXpress, IsoEM, RSEM, Sailfish, and TIGAR2) and above 0.9 for a further three methods (CEM, MMSEQ, rSeq). Both Cufflinks and Scripture performed considerably worse than all other methods, with the corresponding correlation coefficients barely surpassing 0.75. The influence of the library size on accuracy varied somewhat between methods, with the total gain from the sparsest to the richest dataset ranging from approximately 0.01 (Cufflinks) to approximately 0.08 (BitSeq). Out of the nine most accurate methods, MMSEQ appears to be the least sensitive to the influence of read depth (approximately 0.04 gain in accuracy). In order to rule out that our chosen metric for measuring accuracy is prone to producing idiosyncratic results, we have compared it with both the Pearson correlation coefficient and the root mean square error (Additional file 4: Figure S4A). The relative performance of the methods changed only little, indicating that the results were robust with respect to the metric that we chose. Thus, with few exceptions, all methods produce highly accurate transcripts isoform abundance estimates even at moderate read depths.

### Explicit modeling of transcript isoforms leads to more accurate estimation of gene expression levels than count-based methods

Gene expression levels are typically derived from RNA-seq-based data by intersecting the genome coordinates of ‘uniquely-mapped’ reads with the loci of annotated genes and taking into account the length of the transcript that is expressed from a given locus. As may be immediately apparent, this procedure has several limitations. The first is that it is generally unclear what transcript to consider for each locus, when correcting for transcript length. What is typically used is the total length of the ‘union exons’, which is clearly incorrect when the gene expresses multiple isoforms with different relative abundances and different sequences of exons. A second drawback is that the proportion of reads that are discarded depends on the repeat content of the gene with an unknown impact on the accuracy of gene expression estimates. Finally, reads that map across splice boundaries and are informative particularly for estimating the expression of individual isoforms, may be discarded by the simple counting procedure. This problem will preferentially affect expression estimates for genes with a large number of exons and isoforms. Thus, one expects that even gene-level estimates of abundance are improved by the appropriate treatment of transcript isoforms. To test how accurately gene expression levels could be estimated by the benchmarked methods compared to count-based methods, we implemented two variants of count-based gene expression level estimation (‘union exon’ and ‘transcript’-based counting, see Methods). The first method is both simple and widely used, but it has the pitfalls mentioned above. The second method tries to correct some of the inaccuracies of the simple union exon counting method by taking multi-mappers into account and avoiding artificial gene structures. If a method provided gene-level estimates (as is the case for Cufflinks, IsoEM, MMSEQ, RSEM, and rSeq) by default we used these values, otherwise we aggregated estimates of transcript abundances to obtain such estimates. We then compared these gene expression estimates to the true gene expression levels, which were also derived by aggregating the known isoform abundances. When considering only the 12,925 expressed genes (log2 TPM > −5; approximately 26.5 % of all genes), the results (Fig. 2b and Additional file 5: Figure S5) were qualitatively very similar to those that we obtained at the level of transcript expression (Fig. 2a and Additional file 3: Figure S3): estimates of gene expression levels that were produced by or derived from the output of most methods are quite accurate and the accuracy increases with sequencing depth towards saturation. Only BitSeq’s gene-level estimates were strongly sensitive to the size of the input library, in the range of approximately 0.90 for 1 million reads to approximately 0.99 for 30 million reads or more. The same six methods that yielded the most accurate transcript abundances (BitSeq, eXpress, IsoEM, RSEM, Sailfish, and TIGAR2) gave the most accurate gene level expression estimates: all achieved peak Spearman correlation coefficients of 0.98 or higher. CEM, Cufflinks, MMSEQ, and rSeq reached Spearman correlation coefficients of at least 0.95. Scripture, when provided with more than 1 million reads, was also able to estimate gene expression with good (r_s_ >0.9) accuracy. In contrast, the count-based methods only achieved moderate accuracy (maximum r_s_ = 0.89 and r_s_ = 0.86 for the ‘union exon’ and ‘transcript’ methods). As suggested by the scatter plots in Additional file 5: Figure S5, the limited accuracy of either method is largely due to the underestimation of true expression and, as expected, this short-coming is more pronounced in the ‘union exon’ method. As with the transcript estimates, choosing another metric has little impact on the overall ranking/presentation of results (Additional file 4: Figure S4B). Taken together, these results clearly demonstrate that although the accuracy of count-based methods may perhaps benefit from more elaborate procedures for addressing ambiguities in the assignment of reads to loci and transcripts, they still fall short of methods that probabilistically model the generation of RNA-seq data, taking into account transcript isoforms and the sampling of reads from transcripts.

### High expression levels are more accurately estimated than low expression levels

Higher transcript coverage by reads is expected to increase the accuracy with which transcript abundance is estimated. The coverage depends on both the depth of sequencing as well as on the transcript abundance, and indeed we found that the size of the read library has a positive influence on the accuracy of expression estimates. To evaluate the extent to which ‘true’ abundance influences the accuracy of transcript abundance estimates, we grouped both expressed transcripts and genes by their ‘ground truth’ expression into four equally sized bins: low (log2 TPM <0 or 1.1), medium-low (0 or 1.1 < log2 TPM <3 or 4.1), medium-high (3 or 4.1 < log2 TPM <5.5 or 6.2) and high abundance (log2 TPM >5.5 or 6.2), with the first and second numbers referring to the ranges for transcripts and genes, respectively. The overall ranking of tools in terms of their accuracy within expression level bins (Fig. 2c, d) largely reflects what we observed when evaluating the performance on expressed transcripts or genes (Fig. 2a, b). However, the accuracy of transcript expression level estimates degrades progressively from high to low expressed transcripts, with the most drastic drop between the medium-low and low (less than one transcript in 1 million transcripts) abundance (correlation coefficients for the most accurate tools change from approximately 0.75 to approximately 0.4/0.5, at 30 million reads, Fig. 2c). Similarly, estimation accuracies on the gene level differ little across the three bins of most highly expressed genes (mean r_s_ = approximately 0.92, 0.87, 0.85 for the ‘high’, ‘medium-high’, and ‘medium-low’ bins, respectively), but drop most strongly for the bin with the least expressed genes (mean r_s_ = approximately 0.68). Thus, our analysis confirms the expectation that low abundance and, consequently, sparse transcript coverage leads to noisier estimates of expression. However, for genes whose expression levels are in the top three quartiles, the estimates provided by the tools agree very well with the ‘true’ expression levels.

Because different methods appear to handle quite differently transcripts with very low abundance, we sought to further investigate their accuracy in this expression range in particular. More specifically, we determined the rates at which: (1) transcripts or genes that are not expressed are estimated to have non-zero expression (false positive rate); and (2) transcripts or genes that are expressed and are also inferred by a tool to have non-zero expression levels (true positive rate). It should be noted that when dealing with real rather than synthetic datasets, one does not know whether a specific transcript truly had a copy number of 0 in the sample or not. When no evidence of expression is found, some of the Bayesian methods (BitSeq and MMSEQ) strictly assign non-zero ‘prior’ expression probabilities to transcripts, and thus they do not, strictly speaking, produce any ‘false negatives’. Nevertheless, even for these methods it may be relevant to determine how well very limited evidence of expression is handled, and whether transcripts with no such evidence really get assigned ‘prior’ expression values. Thus, after consulting the developers, we have assigned transcripts with an expression estimate which was essentially the method-specific prior value an estimate of zero (see Methods), and then determined the false and true positive rates of all methods. In general, we found that the surveyed methods vary quite considerably in their ability to make accurate ‘present calls’ for transcripts and genes and that tools that exhibit low false positive rates tend to falsely assign zero estimates to a higher fraction of transcripts or genes, as expected (Additional file 6: Figure S6). In this category are IsoEM, RSEM, rSeq, Sailfish, TIGAR2, MMSEQ (‘prior’ expression levels handled as described above), as well as Cufflinks and Scripture (the latter two only when considering gene level estimates). In contrast, CEM, eXpress, BitSeq (zeroed ‘priors’ as described above), Cufflinks, and Scripture (on the level of transcripts), and, in an extreme manner, the unmodified estimates from BitSeq and MMSEQ show the exact opposite behavior. As expected, the rate of true positive calls increases with increasing read depth, as does the rate of false positives. The increase in true positive calls is particularly apparent for lowly expressed genes and transcripts, for which the true positive rate increases steeply up to 30 million reads (Additional file 6: Figure S6E, F). Overall, deeper datasets yield an increased fidelity of making present calls. Consistent with these results, the Spearman correlation coefficients, when calculated across all transcripts and genes (Additional file 7: Figure S7A, B), are considerably lower than when only expressed features are considered (Fig. 2a, b). Given that most of the annotated transcripts were considered ‘not expressed’ in our synthetic dataset, the tools that trade off specificity for sensitivity (BitSeq, CEM, eXpress, MMSEQ) were most affected by the inclusion of not expressed transcripts. Taken together, these analyses indicate that the amount of starting material, the features of interest, and the obtained read depth are all among the factors that influence the accuracy of expression estimates and may play a role in the choice of the method that should ultimately be used for data analysis. Nevertheless, moderate sequencing depth of a few tens of million reads seems to be sufficient for an accurate estimation of most except the very lowly expressed transcripts by many of the available methods.

### The alignment program and bias correction options have little impact on the accuracy of abundance estimates

Some of the surveyed methods strongly recommend the use of a specific short-read alignment program. By default, RSEM even calls such an aligner (Bowtie) internally. Thus, we asked whether the choice of alignment program impacts the accuracy of isoform abundance estimates that are produced by these methods. Surprisingly, we found that the aligner has a relatively small impact on estimation accuracy, regardless of whether one considers transcripts or genes, and only expressed or all features (Additional file 8: Figure S8). If anything, with the exception of CEM, all methods performed better when supplied with read alignments prepared with our custom pipeline that employs the segemehl aligner than when alignments produced by either Bowtie1 (MMSEQ, RSEM) or TopHat2 (Cufflinks, Scripture) were provided. RSEM had the highest gain in accuracy, around r_s_ = 0.05 or r_s_ = 0.03 on the transcript- and gene-level, respectively. On the other hand, CEM produced slightly more accurate results when supplied with TopHat-aligned reads, particularly when considering all features (gain of r_s_ = approximately 0.08). Correspondence with CEM’s developers revealed that the program requires the TopHat-specific SAM/BAM tag ‘XA’, which encodes information about the strand of the transcript to which a read aligns, to correctly parse multi-fragment reads. Because this tag was not supplied in our input alignment files, CEM was unable to properly parse alignments that covered splice junctions and therefore produced less accurate estimates when supplied with our alignments.

A subset of the methods (CEM, eXpress, IsoEM, RSEM, and Sailfish) also attempt to correct various biases that occur during sample preparation, such as positional (non-uniform distribution of reads along transcripts), sequencing (depending on the nucleotide composition of the reads), or mapping (sequencing errors and multi-mapping reads) biases (see Methods section for details). While in general we have restricted ourselves to executing each program with the default parameter settings, we wanted to explore whether bias correction had an impact on the abundance estimation (Additional file 9: Figure S9). Surprisingly, only the transcript estimates produced by CEM and, to a lesser extent, IsoEM were affected. For CEM, the largest difference was observed when considering expressed transcripts, for which bias correction (default: disabled) had a slight detrimental effect (r_s_ loss = approximately 0.05). In contrast, the estimates produced by IsoEM seemed to slightly improve upon enabling the bias correction, but only when all transcripts were considered (r_s_ gain = approximately 0.02). In all other cases, no appreciable differences were observed when executing programs with or without bias correction.

### Gene/transcript structural features affect the estimates of individual methods

**Fig. 3:**
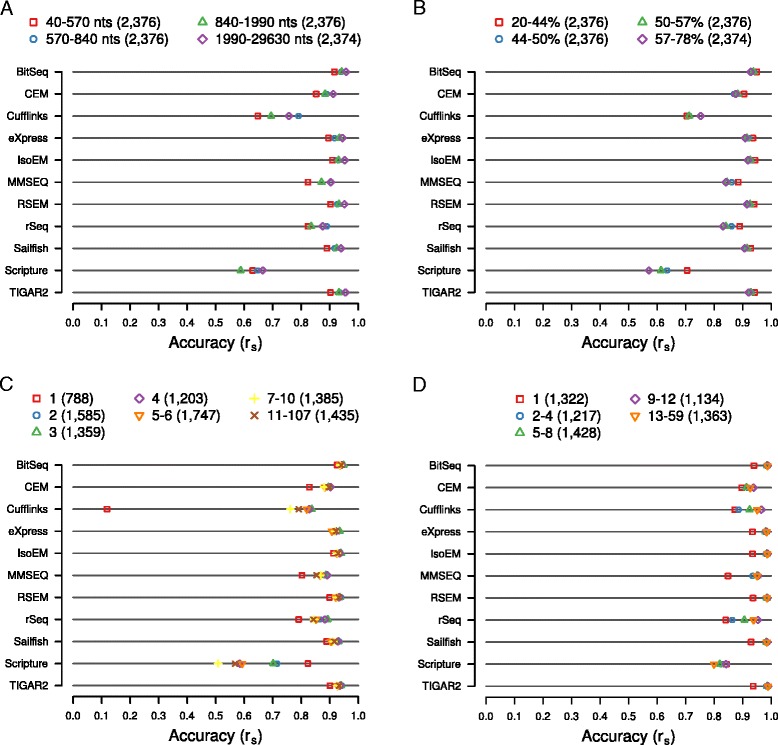
Impact of gene structural features on expression estimates. All transcripts or genes expressed at medium levels (0 < log2 TPM <5.5) were distributed across bins according to transcript length (**a**), GC content (**b**), the number exons per transcript (**c**), and the number of transcripts per gene (**d**). Ranges of the corresponding values covered by each bin are indicated in the legends above each chart. In all cases, expression levels were estimated with each of the indicated methods based on *in silico*-generated sequencing data (read depth = 30 million). The accuracy of estimates was measured in terms of how well they correlate with true expression levels, expressed as the Spearman correlation coefficient r_s_, and is indicated for each bin and method

Next, we aimed to assess the impact of gene structural features on the accuracy of expression estimates. Specifically, we sorted transcripts according to their length, proportion of guanines and cytosines nucleotides (‘GC-content’), and the number of exons of which they are composed. Likewise, we sorted genes by the number of annotated transcript isoforms. Reasoning that the influence of gene structural features on estimation accuracy is likely to be small compared to that of expression level differences, we concentrated on transcripts with mid-range expression, where differences should be most clearly apparent. For each of the structural features, we then defined non-overlapping bins containing comparable numbers of transcripts or genes. Additional file 10: Figure S10 shows the expression level distributions across the different bins for each of the gene structural features. For each bin we then calculated Spearman correlation coefficients between the ‘ground truth’ expression and the estimates produced by each of the surveyed methods when supplied with the 30 million read synthetic dataset (Fig. 3). While none of the analyzed features had a strong and consistent effect on estimation accuracy, we have observed some general trends, as well as method-specific exceptions. The shortest transcripts are quantified with the least accuracy by all methods but Scripture (Fig. 3a). This effect cannot be readily explained by differences in expression level distributions across bins, since the smallest transcripts exhibit, in fact, the highest median expression (Additional file 10: Figure S10A). Moreover, the accuracy of isoform-level estimates steadily increases with transcript length for five of the surveyed methods, with eight methods reporting the most accurate estimates for the longest transcripts. Nevertheless, differences in the correlation coefficients are moderate, in the range of approximately 0.04 (BitSeq) to approximately 0.14 (Cufflinks). Similarly, high GC content appears to have a slight, unfavorable influence on the accuracy of isoform abundance estimates, with all but CEM and Cufflinks producing the least and the most accurate estimates for transcripts with high, and low GC content, respectively, and with the differences in the range of approximately 0.02 (BitSeq) and approximately 0.13 (Scripture) (Fig. 3b). An intriguing phenomenon becomes apparent when analyzing transcripts according to the number of exons that they contain (Fig. 3c): single-exon transcripts are quantified with the least accuracy by all but two methods (Scripture and eXpress). The differences in accuracy relative to bin with the second-lowest accurately are generally small (in the range of approximately −0.01 for BitSeq to approximately −0.05 for CEM) and thus the effect may, at least in part, be explained by the previously described influence of transcript length. However, for Cufflinks this difference is very high (approximately −0.64). Indeed, Cufflinks fails to produce non-zero estimates for the vast majority of single-exon transcripts (Additional file 11: Figure S11A), but not for transcripts containing at least two exons (Additional file 11: Figure S11B, C). This is not due to an incompatibility between Cufflinks and our read processing/alignment procedure, because applying Cufflinks to TopHat2-generated alignments recapitulates the effect (Additional file 11: Figure S11D, E, F). Interestingly, Scripture exhibits the opposite effect, producing the most accurate estimates for single-exon transcripts (difference to next-best bin approximately 0.11). When excluding single-exon transcripts and apart from Scripture, the influence of exon number is marginal, with differences in accuracy across bins in the range of approximately 0.01 (BitSeq) to approximately 0.05 (rSeq).

Similar to single-exon transcripts, genes with a single transcript isoform that generate just one transcript species are least accurately quantified by most methods except Scripture (Fig. 3d). This is to a large extent a consequence of the fact that single-isoform genes are in fact those giving rise to single-exon transcripts (621 of 1,322 genes, that is, approximately 47.0 %). Additionally, genes that have only a small number of associated transcripts also have low expression levels (Additional file 10: Figure S10D). Otherwise, the complexity of the locus appears to have little impact on the accuracy of isoform abundance estimation: maximum differences in accuracy between bins are in the range of approximately <0.01 (Sailfish) to approximately 0.09 (rSeq), with seven methods exhibiting differences below 0.01. Taken together, our results indicate that, apart from a few method-specific exceptions, the influence of gene structural features on the accuracy of estimates is small. BitSeq, CEM, eXpress, IsoEM, RSEM, Sailfish, and TIGAR2 produce the most robust estimates across the assessed features, with the standard deviations of accuracies across the bins analyzed for each feature being around or below 0.025 (Additional file 12: Figure S12). As an additional quantification of the impact of various structural features, Additional file 13 shows the *P* values of the Kolmogorov-Smirnov’s goodness of fit tests carried out for the log-ratio of estimated and expected levels for genes/transcripts in specific bins compared to the entire set of genes/transcripts with moderate expression level (0 < log2 TPM <5.5 and 1.1 < log2 TPM <6.2 for transcripts and genes, respectively; compare categories in Fig. 2c, d).

### Isoform- and gene-level estimates are consistent across biological replicates

**Fig. 4:**
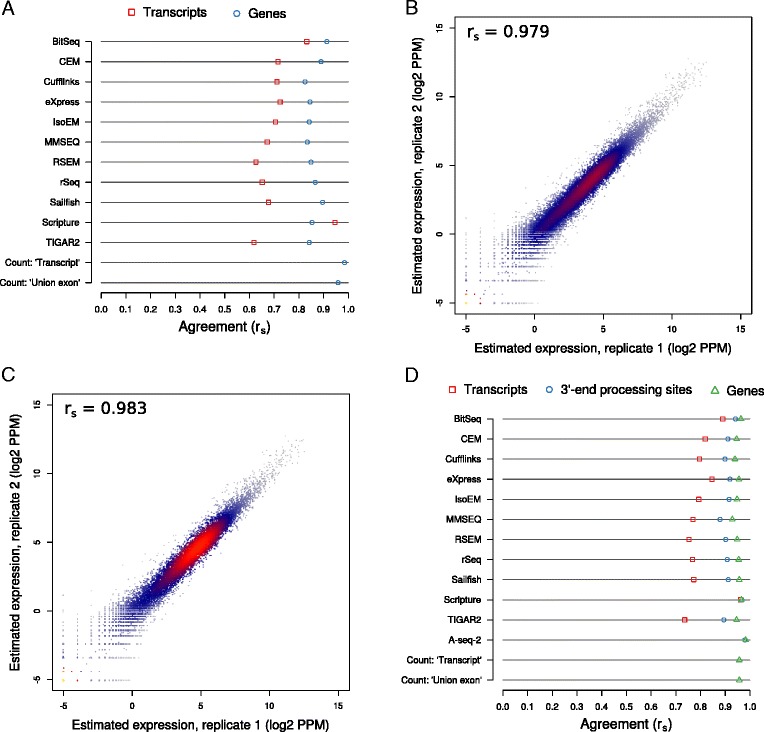
Agreement between expression estimates for replicates of Jurkat cells. **a** Transcript isoform and gene expression levels were estimated with each of the indicated methods from two biological replicates of human Jurkat cell RNA-seq data. The agreement between expression estimates of the two replicates are indicated as Spearman correlation coefficients r_s_, both at the level of transcripts and genes. **b** A-seq-2-based 3′ end processing site expression level estimates for the two replicates are plotted against each other. The Spearman correlation coefficient r_s_ is indicated. **c** As in (**b**), but gene level estimates are compared. **d** As in (**a**), but with the addition of 3′ end processing site abundances. For computing expression estimates for either feature type (transcript, 3′ end processing site, and gene), only those transcripts are considered that end in annotated 3′ end processing sites (see main text and Methods for details)

A basic test for any inference method is whether they produce similar results when supplied with similar data. For isoform quantification, reproducibility was generally tested on data that was generated synthetically. To investigate this aspect, here we have also prepared RNA-seq libraries from two batches of cells of two cellular systems, the murine fibroblast cell line NIH/3T3 and the human T cell line Jurkat. We then supplied the tools for inferring transcript isoform abundances with the resulting short reads (Sailfish) or alignments (all other tools). The replicate agreement, defined as the Spearman correlation coefficient r_s_ between the estimated abundances of (groups of) transcripts in the two human or mouse replicates, was generally high. At the gene level, r_s_ ranged from approximately 0.82 for both human (Cufflinks) and mouse (MMSEQ) to approximately 0.91 (human; BitSeq) and 0.90 (mouse; Sailfish). In contrast, at the transcript level, the agreement was much lower and varied considerably between tools, in the range of approximately 0.62 (TIGAR2) and 0.60 (MMSEQ) to approximately 0.95 and 0.91 (both Scripture) for human and mouse (Fig. 4a and Additional file 14: Figure S13A, respectively). However, only the estimates produced by Scripture and BitSeq showed agreements substantially above r_s_ = 0.7. Most methods produce estimates that are indicative of stronger fluctuations on the transcript compared to the gene level (mean difference in replicate agreement approximately −0.14 and −0.15, for human and mouse), likely because a large proportion of isoforms are expressed at low levels or not at all. In a few cases, differences between replicate agreement on the gene and transcript level exceed 0.2 in at least one species (MMSEQ, RSEM, rSeq, Sailfish, TIGAR2). On the other side of the spectrum, Scripture exhibits a slightly higher agreement between its transcript than its gene level estimates across both organisms (differences of approximately 0.09 and 0.06 for human and mouse, respectively). These behaviors likely reflect differences in the models underlying different methods, particularly with regard to how they treat low abundance transcripts and how readily they assign reads to the minor and major isoforms of a given gene.

### 3′ end sequencing provides independent estimates of isoform abundance

While the tools for inferring isoform abundance have been quite extensively tested on simulated data, obtaining independent and comprehensive experimental reference data is not trivial. Quantitative PCR (qPCR) is the experimental method of choice for the quantification of transcript abundance. However, despite recent technological advances allowing qPCR experiments on a large-scale level, these methods are still cost- and resource-intensive. We therefore applied our A-seq-2 protocol [25] to prepare 3′ end sequencing libraries from the same RNA preparations that were used for RNA-seq and sought to use 3′ end sequencing-based abundance estimates as an independent experimental reference dataset for assessing the accuracy of expression estimates produced by the benchmarked methods.

To assess the quality of these data we first quantified and compared the usage of annotated 3′ end processing sites that overlap the ends of GENCODE-annotated transcripts (see Methods) between biological replicates. We carried out this analysis both at the level of individual 3′ end processing sites as well as at the gene level. For the latter, we aggregated the abundance estimates of all 3′ end processing sites associated with individual genes. Figure 4b (human) and Additional file 14: Figure S13B (mouse) depict the Spearman correlation coefficients between 3′ end processing site abundances across biological replicates, whereas Fig. 4c (human) and S13C (mouse) show the same on the gene-level. In all cases, the agreement was very high (r_s_ >0.97), suggesting that gene expression and 3′ end processing site usage are highly similar in the replicates that we obtained from both human and mouse cells.

Because in constructing the catalog of 3′ end processing sites from published data we applied stringent validation criteria, the set of ‘known’ sites is probably biased towards those that are used in relatively abundant transcripts. We therefore wondered whether the agreement between biological replicates is higher when one focuses only on the GENCODE transcripts that end in a ‘known’, annotated 3′ end processing site and that are likely to be polyadenylated. This was the case for 46,801 human and 26,821 mouse transcripts (corresponding to 25,393 and 17,183 3′ end processing sites, respectively; see Methods section). We selected these transcripts from the output of each method and computed again the correlation between the estimated levels of transcripts, 3′ end processing sites, and genes (the latter two by aggregation; see Methods section) in the two replicates. Figure 4d and Additional file 14: Figure S13D show the results for the human and the mouse datasets, respectively. As expected, the correlation coefficients computed based on transcripts with annotated 3′ end processing sites were, without exception, higher than those computed based on all GENCODE-annotated transcripts (Fig. 4a and Additional file 14: Figure S13A). On the transcript level, Spearman correlation coefficients ranged from approximately 0.74 (TIGAR2) and 0.76 (MMSEQ) to approximately 0.96 and 0.94 (Scripture) for human and mouse, respectively. For 3′ end processing sites and genes, Spearman correlation coefficients of at least 0.88 were reached by all methods for the human and mouse datasets, respectively. The gene expression level estimates provided by the count-based methods also exhibited high agreement (>0.9 for both organisms).

Finally, we further filtered the set of considered transcripts by excluding those whose 3′ ends were not captured in our A-seq-2 dataset. However, in contrast to synthetic data, where the omission of absent transcripts led to a strong increase in estimation accuracy, this did not lead to a further improvement of the correlation between replicate samples (Additional file 15: Figure S14A and B for human and mouse data, respectively). The reasons for this behavior are at the moment unclear. Nevertheless, this analysis indicates that estimates of isoform expression are more reproducible when annotated, and probably more highly expressed poly(A) sites are considered.

**Fig. 5:**
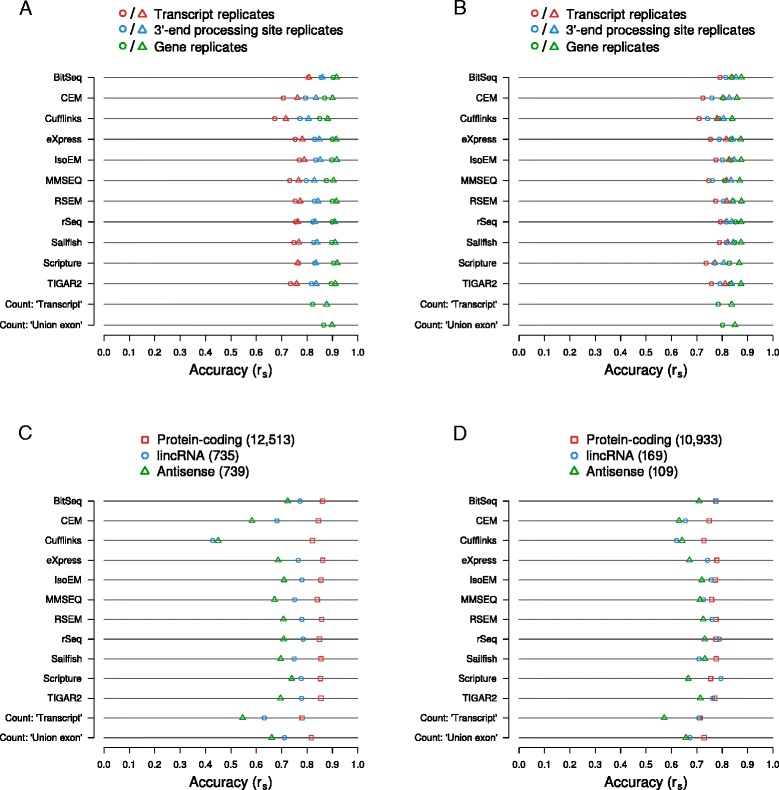
Agreement between the expression level estimated computationally from RNA-seq data and those measured with an independent experimental method. **a** and **b** Abundances of 3′ end processing sites in two independent samples (circles: replicate 1, triangles: replicate 2) of human Jurkat (**a**) or murine NIH/3T3 cells (**b**) were quantified with A-seq-2. Based on RNA-seq data obtained the same cell cultures, the abundances of transcripts ending at these processing sites were estimated with each of the indicated methods and aggregated per processing site. 3′ end processing site estimates were further aggregated per gene. The agreement between A-seq-2- and RNA-seq-based expression estimates was computed as Spearman correlation coefficients (r_s_) for 3′ end processing sites, genes, and transcripts (when processing sites were associated with exactly one transcript). Refer to the main text and the Methods section for further details. **c** and **d** Similar to (**a**) and (**b**), but only gene expression level estimates were considered and Spearman correlation coefficients were computed independently for different classes of gene biotypes, both for the human (**c**) and mouse (**d**) data. Plotted data represent means of the Spearman correlation coefficients calculated for each of two replicates

Having established that the RNA-seq data lead to highly reproducible estimates of isoform expression, we asked whether the computationally estimated expression levels within individual replicates agree with those that were measured experimentally with the A-seq-2 method. As before, we have aggregated the isoform abundance estimates for each 3′ end processing site and these, in turn, for each gene. Moreover, by selecting 3′ end processing sites that overlapped the end of exactly one transcript, we were able to assess estimation accuracy on the level of individual transcripts. As shown in Fig. 5a (human) and 5B (mouse), the expression estimates produced by the surveyed methods are in strong agreement with those based on A-seq-2 across all samples from both human and mouse, with the Spearman correlations approaching those obtained on synthetic data. Agreement between transcript estimates ranges between approximately 0.67 (Cufflinks) and 0.81 (BitSeq) for the human, and approximately 0.71 (Cufflinks) and 0.84 (BitSeq) for the mouse data. When considering 3′ end processing sites that overlapped with the ends of multiple transcripts, correlations further improve, with Spearman correlation coefficients for human and mouse data now in the range of approximately 0.77 (Cufflinks) to 0.86 (BitSeq), and approximately 0.85 (BitSeq) to 0.74 (Cufflinks) respectively. For reference, the corresponding scatter plots for the first replicates of each dataset are presented in Additional file 16: Figure S15 (human) and Additional file 17: Figure S16 (mouse). Finally, aggregation of 3′ end processing site estimates per gene led to a further increase in agreement by approximately 0.04 to approximately 0.08 in both organisms. Assuming the A-seq-2-based estimates of expression as ‘ground truth’, Scripture (r_s_ = approximately 0.92) and RSEM (r_s_ = approximately 0.88) delivered the most accurate estimates at the gene level for human and mouse data, respectively. Importantly, we found that even when estimating gene-level abundance from biological data, isoform-aware methods yield more accurate results than the broadly used count-based methods. Across all methods, level of coarse-graining, and organisms, the second replicate yields estimates that are slightly more accurate, likely reflecting a batch effect pertaining to the preparation of RNA-seq and A-seq-2 sequencing libraries. On all levels, differences in accuracy between most methods are rather small, similar to what we observed on synthetic data. Also similarly, enabling or disabling bias correction in those methods that provide such an option also did not substantially alter the accuracy of estimates on experimental datasets (Additional file 18: Figure S17) and in the case of CEM, we have observed a consistent detrimental effect of bias correction across transcripts, 3′ end sides, and genes, and in both organisms.

As a practical guideline for those researchers studying non-coding genes, we also wondered how accurately the surveyed methods can quantify the expression of different classes of genes. Therefore, we computed the agreements of expression estimates with those inferred with A-seq-2 on genes annotated as ‘protein coding’, ‘lincRNA’ (long intergenic non-coding RNAs), and ‘antisense’ in both human and mouse. For human, the 12,513 protein coding genes amenable to quantification by A-seq-2 are considerably more accurately quantified than lincRNA (739) and antisense genes (739), with Spearman correlation coefficients reaching values of up to approximately >0.85, 0.8, and 0.7, respectively, for the different gene classes (Fig. 5c). The absolute difference in estimate accuracy across these classes is particularly striking for Cufflinks, where the Spearman correlation coefficients are reduced by almost 0.4 when trying to estimate lincRNA or antisense RNAs rather than protein coding genes. This may reflect the issue that Cufflinks seem to have with quantification of single-exon transcripts. Given the differences in median A-seq-2-based expression levels across each gene class (log2 PPM = approximately 3.73, −0.63, and −0.49 for protein coding, lincRNA, and antisense genes and considering both replicates), it is likely that the observed differences in estimation accuracy are, at least in part, a function of the true expression levels of these genes. Although the general trend is the same across the mouse samples, the differences in estimation accuracies between the different gene types are not as pronounced as in human, and for some methods the quantification of lincRNA genes is actually more (rSeq, Scripture) or approximately equally accurate (BitSeq, TIGAR2) as that of protein coding genes (Fig. 5d). This may reflect the true abundance of these genes because A-seq-2 estimates of the median expression of lincRNA and antisense gene classes were somewhat higher for mouse (log2 PPM = approximately 0.00 and 0.10, respectively) while those for protein coding genes were about the same (median log2 PPM = approximately 3.67). Taken together, the estimates of isoform expression based on biological data and evaluated against expression measurements obtained with an independent experimental method validate and recapitulate the most important conclusions derived from the synthetic data: many of the surveyed methods are able to estimate isoform abundances with good accuracy, particularly when true expression levels are high. Furthermore, employing any of these tools improves the accuracy of gene expression level estimates relative to widely used count-based methods.

## Discussion

Accurate quantification of gene expression is essential for the understanding of gene regulatory processes in health and disease. Due to its large dynamic range, high reproducibility, and the ability to detect previously unknown transcripts, RNA sequencing has become the method of choice for global expression profiling. However, despite the digital nature of the resulting data, technical limitations (limited read length and non-uniform transcript coverage) render their analysis challenging, especially when large and complex genomes of higher eukaryotes, with frequent repeats and overlapping gene structures, are involved. Accurate computational methods for RNA-seq data analysis therefore remain in high demand. This is reflected in the large number of computational methods for estimating transcript isoform abundance that were developed over the course of the last 6 years. Naturally, the question arises which method should best be used in a particular context. Here we have tried to address this question in depth, using not only synthetic data, as is typically done when the computational methods are developed, but also using estimates that were obtained with an independent experimental method for a specific type of isoforms, namely those that arise from alternative polyadenylation. This is because methods for global quantification of 3′ end site usage distinct from RNA-seq are available [25, 29–31] and have been used quite extensively to analyze changes of 3′ UTR isoforms across conditions. A drawback of these methods is that they cannot distinguish between transcripts that are processed at the same poly(A) site. However, although most mammalian genes have multiple poly(A) sites, currently, over 60 % of the poly(A) sites whose expression we have been able to measure with A-seq-2 have only one associated transcript annotated in the human or mouse GENCODE datasets. Thus, we believe that A-seq (or another method for quantifying the usage of 3′ end processing sites) can offer a good alternative to qPCR as a comprehensive approach to transcript isoform quantification. Nevertheless, as the 3′ end sequencing protocols are relatively new, it is likely that the computational analysis of these data can be further improved.

Expecting that most users would – at least initially – run the methods ‘out-of-the-box’, we sought to apply the surveyed methods with default settings, and departed from this general rule only to test the influence of specific options that the developers of the methods proposed. Although we found that neither the use of recommended short read aligners nor the activation of bias correction generally improved estimation accuracy, it is likely that the developers of the individual methods or experienced users would be able to improve the performance of individual tools in specific settings. During the course of this study we discovered a number of assumptions that the programs tacitly made and that affected the interpretation of the results. Therefore, a specific recommendation that we can make to developers is to ensure that sufficiently detailed information on input requirements, potential pitfalls and the implication of specific options (ideally including usage examples) is provided.

Encouragingly, we found that most of the methods that are currently used to estimate transcript isoform abundance produce quite comparable and accurate results, both on synthetic and experimental data. As a general trend, methods such as Scripture and Cufflinks, whose main objective is to assemble/reconstruct transcript isoforms but that have also been co-opted for estimating isoform abundance, perform poorer than methods specifically designed for the latter purpose. However, such methods could be part of the initial assembly of a comprehensive set of transcripts whose expression can be subsequently quantified with a different approach [17]. Cufflinks is part of the popular ‘Tuxedo Suite’ pipeline (Bowtie-TopHat-Cufflinks) and for the purpose of inferring isoform abundances from RNA-seq data is probably superseded by the eXpress method developed by the same group [32]. Importantly, the gene level expression estimates obtained by cumulating the abundances of transcript isoforms inferred with almost any of the surveyed methods are more accurate than those produced by ‘count-based’ methods that are widely used in the analysis of gene expression. This is likely because count-based methods either disregard or mis-assign reads whose origin (genomic locus or isoform) cannot be unambiguously determined. We therefore strongly advise to use methods for transcript isoform quantification (such as those benchmarked here) even when only quantification at the gene level is desired.

Next to a general assessment of the accuracy of expression estimates produced by the tools, we also studied the impact of several transcript properties on the accuracy of expression estimation. We found that parameters that directly influence the coverage of a transcript or gene by reads, such as sequencing depth and true expression level, have a positive influence on estimation accuracy, as has been observed before [33]. On synthetic data, disregarding features that are not expressed led to a strong increase in the accuracy of expression estimates, particularly on the level of isoforms. Thus, as may be expected, estimates of low-abundance isoform expression are not very reliable. How isoforms that are expressed at very low levels (or not at all) are treated in practice, varies between methods. Most methods report (or imply) cases of ‘zero’ expression and some allow the user to specify a minimum level of expression for reported transcripts. On the other hand, BitSeq and MMSEQ do not enforce such a threshold and instead attempt to assign non-zero priors even to transcripts that are not supported by any read, based on factors such as the library size and transcript length. These solutions represent lower and upper bounds on the expression of low-abundance transcripts (in contrast to higher-abundance transcripts, for which precise estimates of expression are sought). In typical RNA-seq experiments, where many transcripts are expected to be expressed, how precisely absent transcripts are treated may not be essential. However, in the case of, for example, single cell sequencing, the proportion of annotated transcripts that are not detected can be quite large and one should be aware that the meaning of the expression values that the programs report are not entirely the same for expressed and not expressed transcripts. Next to coverage-related factors, we found that the length and GC content of transcripts as well as the complexity of the gene locus (exons per transcript and transcripts per gene) have a small impact on the accuracy of inferred expression levels, which is probably of more interest to the developers rather than to the average user.

To ensure the widest applicability of our findings, we have based our study on single-end, short read (50 nt) data. Illumina’s paired-end sequencing technology, which has been employed in previous comparisons of isoform abundance estimation methods [21, 33], provides additional information that may be used by many of the evaluated methods to improve the assignment of read fragments to the correct isoform and thereby the accuracy of abundance estimates. As has been previously demonstrated [33–35], increasing the read length should also enhance the accuracy of abundance estimates, because it leads to a reduction in the fraction of reads that cannot be unambiguously assigned to the correct isoform. Indeed, increasing the read length is a current trend in the field of next generation sequencing. For example, Pacific Biosystems technology now allows full-length transcript sequencing [15], although at limited throughput.

While most methods produce comparable and fairly accurate estimates of transcript isoform abundance, they differ more strongly in their computing needs. In some cases, speed comes at the cost of increased memory requirements, which is evident for example with IsoEM, which is extremely fast, but uses tens of GB of memory. Nonetheless, with the increase in the number and size of the datasets that one typically analyzes, speed and scalability of processing become very important considerations for the utility of a program. The recently developed Sailfish is of particular interest in this regard because its running times scale well within the tested range of sequencing depths, while the memory footprint remains reasonable. Moreover, its alignment-free k-mer-based approach disposes of the time-consuming step of aligning reads to a reference genome or transcriptome. For typical datasets of approximately 100 million reads, most programs use 1–20 GB of memory and run for 1–2 h. An exception is TIGAR2, which produces highly accurate expression estimates that come at the cost of both high running times and high memory use.

One important aspect that was beyond the scope of the current study is that in many studies, the interest is the identification of transcript isoforms that are differentially expressed between two conditions, rather than the quantification of isoform abundance in a specific condition. The estimates of isoform abundance inferred with the methods that we tested here can in principle be used in subsequent statistical tests for differential expression, but the issue of the underlying model has not been entirely addressed. If sufficient replicates are available, two-sample parametric or non-parametric tests can be used. However, due to the high costs of RNA-seq experiments, the availability of more than a few replicates is very rare. Instead, when the number of replicates is small, accurately accounting for the different sources of variability in the data is important. Differential expression analysis based on RNA-seq data is frequently done with programs such as baySeq [36], DEGSeq [37], DESeq [38], or edgeR [39] (reviewed in [40]). These programs work on (integer) count data and use specific models for the number of reads that are expected from individual ‘features’ such as exons or genes. Therefore, they are not appropriate for the estimate of transcript abundances that are obtained with the programs that we analyzed here. Fortunately, some of the evaluated programs have additional modules for differential expression analysis. BitSeq has a built-in functionality for differential expression analysis based on the transcript expression levels estimated by the tool. The developers of Cufflinks and eXpress suggest Cuffdiff [41] for gene and transcript differential expression based on their respective outputs. The developers of IsoEM suggest the bootstrapping-based IsoDE [42] for differential expression analysis, but this tool is restricted to comparisons at the gene-level only. MMSEQ’s developers suggest MMDIFF [43] which performs model comparisons and takes as input the posterior summaries from the MMSEQ tables. Alternatively, they provide instructions to feed MMSEQ-estimated counts to count-based differential expression analysis tools like DESeq or edgeR [44]. eXpress and Sailfish developers also suggest to feed the supplied (rounded) ‘effective counts’, and ‘expected number of reads’, respectively, into one of the count-based differential analysis tools mentioned above. Finally, RSEM developers suggest EBSeq [45], a Bayesian differential expression analysis method for genes and isoforms across two or more biological conditions. EBSeq is integrated into the RSEM suite [46].

## Conclusions

In summary, several methods for the inference of transcript isoform abundance can accurately quantify expressed transcripts even from relatively small short-read libraries and should thus be adequate for the analysis of both past and present RNA-seq datasets. Their performance is largely not affected by structural features (number of exons, transcript length, GC content) of the genes/transcripts, although, as expected, abundant transcripts are quantified more accurately compared to rare transcripts. Importantly, our analysis indicates that the explicit quantification of transcript isoforms leads to more accurate estimates of gene expression levels compared to the ‘count-based’ methods that are broadly used currently. Given the wealth of tools available, the user can largely base his choice of method on criteria related to usability, available processing and memory capacities, compatibility with pre-existing data processing pipelines, and the desired downstream analyses (see Table 2). Especially promising is the most recently proposed approach that relies on k-mer frequencies, bypassing entirely the read-to-genome/transcriptome alignment and thereby enabling analysis of very large collections of samples, such as those that have started to emerge from patient studies. Developers may profit from our study setup, particularly our efforts to provide compatible datasets to tools with quite different requirements as well as our approach at validating estimation accuracies of a particular type of isoform with an independent large-scale experimental method. We propose that methods such as 3′ end sequencing and cap analysis of gene expression (CAGE; [47]), which allow quantification of alternative polyadenylation and transcription start sites, respectively, could complement validation schemes based on synthetic data and quantitative PCR in future or ongoing assessments of RNA-seq analysis methods, such as, for example, by the MAQC-III/RNA-C consortium [48].

**Table 2:** Features and performance summary of the surveyed methods

Method	Extensive documentation	Standard file formats	Gene-level estimates	Reconstruction supported	DE analysis	Efficient multi-threading	Fast	Small memory footprint
BitSeq	X	X			X	X		
CEM		X		X			X	X
Cufflinks	X	X	X	X	X	X		
eXpress	X	X			X			X
IsoEM		X	X			X	X	
MMSEQ	X	X	X		X			X
RSEM	X		X		X	X		
rSeq	X		X					X
Sailfish	X	X			X	X	X	X
Scripture	(X)^*^	X		X				
TIGAR2	X	X						

To facilitate a user’s choice of method, we indicate which methods meet various criteria of usability, functionality, and performance, as follows: ‘Extensive documentation’ - documentation that goes beyond the description of parameters is provided (document, web page, FAQ which allowed us to run a given method confidently and without help from developers); ‘Standard file formats’ - the method exclusively operates on the indicated file formats for transcript sequences (FASTA), gene/transcript annotations (GFF/GTF or BED12), read sequences (FASTA or FASTQ), and read alignments (SAM/BAM as defined in [65] and produced by most modern aligners); ‘Gene-level estimates’ - estimates of expression on the gene level are provided in addition to those at transcript level; ‘Reconstruction supported’ - the method can also reconstruct transcript models based on the provided sequencing/alignment data; ‘DE analysis’ - the developers make a general recommendation or provide an integrated solution for differential analysis of transcript/isoform expression; ‘Efficient multi-threading’ - the method efficiently makes use of multiple cores (speedup of at least two-fold in at least three out of five datasets; see Additional file 2: Figure S2A); ‘Fast’ - processing of 100 million synthetic reads or their corresponding alignments completed in less than 1 h (16 cores and 64 gigabytes provided; see Fig. 1b); ‘Small memory footprint’ - all synthetic datasets could be processed with less than 8 gigabytes of memory (independent of the number of cores used; see Fig. 1c, d). Additional details are provided in the main text. *The documentation for the complete Scripture suite is extensive, but a detailed description of the archive ‘ScriptureScorer.jar’ that contains only the RNA-seq quantification module which we used here is not available. Furthermore, the options for this module are different from those described for the main program.

## Methods

### Genomes, gene annotations, and transcriptome sequences

The hg19 (human) and mm10 (mouse) genome assemblies were obtained from UCSC Genome Bioinformatics, University of California, Santa Cruz [49]. Haplotype chromosome versions were discarded. Releases 19 and M2 of the GENCODE gene annotation sets GENCODE [50] were used for the analysis of human and mouse data, respectively. Version numbers were stripped from gene and transcript identifiers. In the human annotation set, all features on the Y chromosome that are present, in identical form, on the X chromosome have gene identifiers of the form ‘ENSGRx’ (with x being a 10-digit number), and the corresponding features on the X chromosomes have identifiers of the form ‘ENSG0x’. We discarded the former to avoid essentially duplicate features. Sequences of annotated transcripts (‘transcriptomes’) were obtained from ENSEMBL (release 74, compatible with GENCODE v19 and vM2) [51]. Genome and transcriptome sequences in FASTA format were indexed with segemehl [52].

### Generation of synthetic sequencing data

To generate *in silico* reads, we have used the Flux Simulator software [26], with the hg19 genome and GENCODE v19 annotation set processed as described above. Because we focused on the quantification of long RNAs, we further removed from the annotation set, all entries whose gene or transcript type attributes matched either ‘miRNA’, ‘misc_RNA’, ‘rRNA’, ‘snoRNA’, ‘snRNA’, ‘Mt_rRNA’, or ‘Mt_tRNA’. Taking into account the annotated transcripts introduced above as well as a target number of transcript molecules (we chose 5 million), Flux Simulator randomly assigns expression ranks to transcripts according to Zipf’s Law. The software then attempts to model the various steps in a typical RNA-seq library preparation protocol, including fragmentation, reverse transcription, and PCR amplification, to generate reads. We ran Flux Simulator with the options --express, --library, and --sequence. Additional parameters were supplied in a parameter file (Additional file 19) as outlined in the Flux Simulator manual [53]. Flux Simulator does not natively support generation of directional single-end read libraries. To obtain these, we instead generated a pool of 692,414,670 paired-end reads from which we then discarded all antisense mate sequences, as suggested by the Flux Simulator developers. To facilitate downstream processing, the identifiers of the remaining reads were simplified and their sequences capitalized. Identical read sequences were collapsed with the fastx_collapser [54]. Finally, poly(A)-tails - introduced in the simulation - were removed with the cutadapt software [55] by specifying a stretch of 50 adenines as the 3′ adapter and the non-default options --overlap=1 and --minimum-length=15. This resulted in a set of 298,435,172 poly(A)-free, directional, single-end reads. From this initial set, we randomly selected, progressively, approximately 100 (100,001,950), 30 (30,004,152), 10 (10,000,760), 3 (2,998,971), and 1 (999,436) million reads to analyze the scaling behavior of the programs.

### Preparation of sequencing libraries

Human Jurkat T lymphocytes (ATCC TIB-152) [56] and NIH/3T3 mouse embryonic fibroblasts (ATCC CRL-1658) [57] were cultured in RPMI medium (Sigma) at 37°C and 5 % CO2. Cells were collected at approximately 70 % confluency after trypsinization. 3′ end libraries were generated by the A-seq-2 protocol, which captures sequences immediately upstream of mRNA 3′ end processing sites and poly(A)-tails [58], and directional RNA-seq libraries were prepared according to the Illumina-provided protocol. For both protocols, poly(A)-positive RNA was isolated from the cells with the ‘Dynabeads mRNA DIRECT Kit’ (Ambion) and fragmented by alkaline hydrolysis to fragment sizes of 150–300 nt. Following reverse transcription and PCR amplification, the libraries were sequenced single-end with a read length of 51 nucleotides on an Illumina HiSeq-2000 platform.

### Pre-processing of human and mouse RNA-seq data

Potential 3′ adapter and poly(A)-tail fragments were sequentially removed from FASTQ-formatted short reads sequences with two iterations of cutadapt [55], specifying the 3′ adapter sequence and a stretch of 50 adenines, respectively, to the --adapter option. Other non-default options were --overlap=1 and --minimum-length=15. Identical sequences were collapsed with the fastx_collapser [54].

### Alignment of synthetic and experimentally obtained reads to genomes and transcriptomes

The experimentally obtained sequence sets, as well as the five *in silico*-generated read subsets (FASTA-formatted), were aligned to the genome and transcriptome of the respective species with segemehl 0.1.7 [52], with default parameters (minimum percentage of matches: 90 %) and without using the spliced alignment option. Anti-sense alignments to transcripts were discarded from further analysis. For the surveyed methods that require input alignments in ‘genome space’, transcriptome alignments were converted to genomic coordinates with custom scripts based on the gene models provided in the GENCODE v19 annotation file. Directly and indirectly obtained genome alignments in SAM format were merged, duplicate alignments resulting from the conversion between transcript and genome coordinates were discarded, and the remaining alignments were filtered such that for each read only the alignments with the smallest edit distance were kept. For methods requiring input alignments in ‘transcriptome space’, the transcriptome alignments of each reads that had an edit distance larger than the minimum distance obtained in aligning the read to the genome were discarded.

During the course of the study, we have noticed that the transcript isoform quantification methods that we evaluated make certain assumptions about the format of the input alignment files and that in some cases these assumptions only hold for certain short read aligners or for outdated file formats. We therefore implemented additional post-processing steps to ensure that the information required by individual programs is present in the alignment file is the appropriate form. (1) We ‘uncollapsed’ the reads: across all alignment files, alignments corresponding to collapsed reads were ‘cloned’, but a randomized QNAME name was assigned to each individual read that was only re-used for additional alignments of the same read. (2) To avoid misinterpretation of tag fields, all custom segemehl tags were removed. (3) Reads aligning to more than one reference locus are reported by segemehl as individual alignment records with identical read names (QNAME field). In accordance with the SAM specifications [59], we have further added a linked-list encoding for such reads. Specifically, we have designated the first out of such a group of alignments as the primary (0x100 bit of the FLAG field unset) and introduced CC and CP tags, pointing, respectively, to the reference sequence name and the starting position of the following alignment. All remaining alignments were designated secondary (0x100 bit set), and CC and CP tags were added to all alignments but the last in the list. Moreover, the HI (0-based ‘hit index’) tag was added to all alignments of ‘multi-mapping’ reads. The NH (‘number of hits’) tag was re-computed for all reads in a given alignment file. (4) segemehl reports a default mapping quality (MAPQ) of 255 for each alignment record. Following the example of TopHat2 [60], we have reset the mapping quality values based on the number of alignments reported for a given read. Specifically, we have assigned mapping qualities of 50 (NH = 1), 3 (NH = 2), 1 (NH = 3 or 4), and 0 (NH = 5 or more). (5) We introduced sequencing quality strings (QUAL field). For *in silico*-generated reads, which did not have such scores associated, strings of ‘I’ characters that match the length of the read sequence (SEQ field) were used to denote maximum quality scores (according to the Sanger FASTQ format). In the case of the experimental RNA-seq libraries, we used the quality scores that were provided in the initial FASTQ files that were obtained from the sequencing facility. The data processing was automated with the help of the Anduril [61] data analysis framework. To test the influence of the alignment program, we have also generated alignments of *in silico* generated reads with Bowtie (version 1.0.0) [62] and TopHat2 (version 2.0.10) [60]. The output of these programs were used without further processing.

### Analysis of 3′ end sequencing data

The reads obtained with the A-seq-2 protocol for 3′ end sequencing have a particular structure: they are the reverse complement of 3′ end RNA fragments and further have the sequence AAANNNN downstream of the actual 3′ end [58] for details). To recover the mRNA 3′ ends from these sequenced reads, we therefore first trimmed the expected NNNNTTT sequences from the 5’ ends of the reads, removed the 3′ adapter with the removeAdaptor.pl function of the CLIPZ server [63] and kept only sequences longer than 15 nt. We reversed complemented the sequences and mapped them to the corresponding genome and transcriptome with segemehl v0.1.7 [52] and default parameters. Next, we transformed transcriptome alignments to genomic coordinates, merged them with the genome alignments, discarded duplicates and kept for each read only those alignments with the smallest edit distance (see above). Finally, we collapsed the 3′ ends of the aligned short reads and produced a BED file recording the exact genomic positions of 3′ end cleavage together with the aggregated read counts. For reads that mapped to multiple loci in the genome, counts were equally distributed across loci. As we and others observed before, 3′ end formation appears to occur with a certain degree of microheterogeneity, that is, prominent 3′ end sites are usually being flanked by less frequently used 3′ end sites. Because these latter sites may not reflect functional biological variation, closely spaced 3′ end sites are typically clustered into 3′ end processing regions [25]. Many 3′ end sequencing protocols capture sequences that result from priming at internal adenosine stretches rather than poly(A)-tails at the step of cDNA synthesis. To exclude a protocol-specific bias in 3′ end quantification, we only analyzed 3′ end processing sites that are supported by at least two independent 3′ end sequencing protocols. These are annotated in our in-house polyAsite database (manuscript in preparation) [64]. For each 3′ end processing region, we determined the number of overlapping A-seq-2-inferred 3′ end reads, which we used as a measure of the expression of the corresponding 3′ end processing region. In total, we quantified the expression of 90,128 and 61,457 3′ end processing regions in human and mouse, respectively.

### Estimation of transcript isoform abundance

With the exception of Sailfish (see below), all of the programs compared in this study use alignments of reads to either the transcriptome or the genome. We used the samtools [65] suite to sort or interconvert the SAM/BAM alignment files obtained from mapping reads to genomes/transcriptomes. To detect and quantify ‘multi-mapping’ reads, several methods require that the alignment files are ordered such that the alignments of a given read occur one after the other. Additionally, some methods further require that reads that are similar in sequence (and their associated alignments) are randomly distributed in the input file. This is of clear relevance for eXpress, which processes alignments ‘on-line’ and trains its parameters from the data. In such cases, ‘non-random’ occurrence of the read alignments may lead to biased parameters and output. Typically, both of these conditions (reads occur in random order while all alignments of a given read are grouped together) are met when alignments are sorted by the names of the reads, which is recommended in the documentation of these methods. But if the pre-processing pipeline includes sorting and renaming steps (for example, collapsing and uncollapsing of reads with identical sequences), sorting the alignment file by read names may lead to a situation in which neither condition is fulfilled. Unfortunately, the precise assumptions about the order in which read alignments should appear in the input file are not typically mentioned in detail in the documentation of the programs. We thus recommend that users ensure that the order in which reads appear in the alignment file that is used as input to an isoform quantification method is ‘randomized’ whenever the quantification method recommends sorting alignments by read name.

Scripture and CEM require annotation files in a BED-based format which supports multiple fragments (that is, exons) per entry and is known as BED12 or BED12+3. These were generated from the GENCODE-provided GTF annotation files with the help of the R/Bioconductor package rtracklayer [66]. Because some methods required the mean and standard deviation of the fragment/read length distribution, we calculated these from the alignment files with a custom script. In the following, the steps taken to execute each surveyed program are outlined.

BitSeq [67, 68] uses as input transcript sequences in FASTA format and alignments of reads to the transcriptome in SAM or BAM format, sorted by read name (randomized). We have used the command-line version of BitSeq (version 0.7.5), but an R/Bioconductor version is also available.

The first step in BitSeq is to parse the alignment file to calculate probabilities of individual reads originating from individual transcripts:

- parseAlignment \ <alignments_transcriptome> --trSeqFile \
- <sequences.fa> --outFile \ <out_prefix.prob> --trInfoFile \
- <out_prefix.trx> --uniform \

Then the mean transcript expression is estimated with a Variational Bayes inference algorithm:

- estimateVBExpression <out_prefix.prob> \ --outPrefix <out_prefix> \
--outType RPKM --trInfoFile \ <out_prefix.trx> --samples 1000 --seed 1 \

By default, when no read alignments are assigned to a given transcript, BitSeq sets the expression estimate of the transcript to a ‘prior’ that depends on the effective transcript length and the sequencing depth. When indicated and in communication with the developers, we have identified these cases by finding transcripts for which the expected read count (alpha parameter of the Dirichlet distribution) equals exactly 1 and replaced their RPKM estimates with zeros.

CEM [69] takes as input a BED12 file of transcripts and a SAM or BAM file of genomic alignments, sorted by genomic coordinates. We ran CEM (processsam version 2.5.2) as follows:

- python runcem.py --annotation \ <annotations.bed12> --forceref \
--prefix <out_prefix> \ <alignments_genome.bam> \

Where indicated, we have set the --usebias option to evaluate CEM’s built-in bias correction functionality, which attempts to correct for positional, sequencing, and mappability biases.

Cufflinks [70] takes as input an annotation file in GTF format and a SAM or BAM file of read alignments to the genome, sorted by genomic coordinates. We ran Cufflinks version 2.1.1. with the following command:

- cufflinks --GTF <annotations.gtf> \--library-type fr-secondstrand \
--frag-len-mean <fragment_length_mean> \ --frag-len-std-dev \
- <fragment_length_sd> --multi-read-correct --output-dir <out_dir> \ <alignments_genome.bam> \

Only expression estimates with ‘fpkm_status’ ‘OK’ were considered. All other estimates were set to zero.

eXpress [32] takes as input a FASTA file of transcript sequences and a SAM or BAM file of transcriptome alignments, sorted by read name (randomized). We ran eXpress version 1.5.1. with the following command:

- express --no-update-check --f-stranded \ --frag-len-mean \
- <fragment_length_mean> --frag-len-stddev <fragment_length_sd> \
--output-dir <out_dir> <sequences.fa> \
- <alignments_transcriptome.bam> \

As eXpress is correcting for biases introduced during library preparation (specifically, fragmentation and priming) by default, we have set the --no-bias-correct option when evaluating the performance of methods without bias correction.

IsoEM [71] takes as input a GTF file with transcript annotations and a SAM file of genomic alignments, sorted by read name (randomized). We obtained instructions for running IsoEM from [72] and ran the program (version 1.1.1) as follows:

- isoem --GTF <annotations.gtf>--\fragment-mean \
- <fragment_length_mean> --fragment-std-dev \
- <fragment_length_standard_deviation> \--directional -o <out_file> \
- <alignments_genome.sam> \

IsoEM also attempts to correct for fragment sampling biases resulting from random hexamer priming during reverse transcription and to evaluate this functionality, we have generated isoform abundance estimates with the -b option.

MMSEQ [73] (version 1.0.8) takes as input a file with transcript sequences in FASTA format as well as a BAM file with read alignments to the transcriptome, sorted by read name (randomized). We ran MMSEQ based on the provided instructions [74]. In particular, we first mapped reads to transcripts:

- bam2hits <sequences.fa> \ <alignments_transcriptome.bam> > <hits> \

and then obtained expression level estimates via:

- mmseq <hits> <out_prefix>

Note that unlike all other methods, MMSEQ does not report RPKM values, but rather the means μ of the posterior isoform expression distributions. As these are reported as log (base e) values, we first exponentiated them for our analyses. Similar to BitSeq, MMSEQ defaults to assigning ‘prior’ expression estimates to those transcripts for which no read/alignment evidence can be found. Where indicated, and in communication with the developers, we have identified such cases by substituting in MMSEQ’s output the log μ estimates for all transcripts or genes with a ‘unique_hits’ count of 0 with ‘NA’.

RSEM [34, 35] (version 1.2.18) works on alignments of reads to transcripts (sorted by read name/randomized in SAM or BAM format). Based on GENCODE annotations, we first generated a tab-delimited lookup table between ENSEMBL gene (first field) and transcript IDs (second field). For each organism (human or mouse), we then generated RSEM-specific indices of the corresponding ENSEMBL transcript sequences (FASTA) with the following command:

- rsem-prepare-reference --no-polyA--transcript-to-gene-map <gene_id_transcript_id_table> <sequences.fa> <index_prefix> \

RSEM requires read alignments to the transcriptome. However, because the tool cannot process alignments that contain insertions or deletions (indels), we purged the alignment file of any entries that contained disallowed characters in their CIGAR string fields (D, H, I, N, P, S). After recalculating read length distributions across the resulting alignment files, we estimated maximum likelihood expression levels as follows:

- rsem-calculate-expression --sam --strand-specific --no-qualities \
--seed-length 15 --fragment-length-mean <fragment_length_mean> \
--fragment-length-sd <fragment_length_sd> \
- <alignments_transcriptome.sam> <index_prefix> <out_dir>

To evaluate RSEM’s built-in bias correction functionality, which attempts to correct protocol-specific 5′ or 3′ positional biases, we have set the --estimate-rspd (read start position distribution) option where indicated.

rSeq [75] takes as input a FASTA file of transcript sequences and a SAM file with read-to-transcript alignments, sorted by transcript names and coordinates. Because the header for each transcript in the sequence file is expected to be of the form ‘gene_id$$transcript_id’, we used custom scripts to construct these identifiers and substitute the reference sequences in the sequence dictionary and alignment entries of the transcriptome alignment file accordingly. We then obtained rSeq-based (version 0.2.0) isoform expression levels with the following command:

- rseq expression_analysis <sequences.fa> \
- <alignments_transcriptome.sam>

Sailfish [76] (version 0.6.3) takes as input transcript sequences in FASTA format and sequenced reads in FASTQ (or FASTA) format. Sailfish does not required reads to be ordered in a specific manner. The first step in running Sailfish is to index the transcriptome sequences:

- sailfish index -t <sequences.fa> -o \ <index> -k 20 \

and then the isoform abundance estimates are obtained with the following command:

- sailfish quant -i <index> -l T=SE:S=S -r \ <reads> -o <output_prefix> \

Sailfish considers transcript length, GC content, and dinucleotide frequencies as possible sources of bias and uses a regression model to correct for them. By default, Sailfish reports its output both with and without these ‘bias correction’ settings. Unless otherwise noted, we have used the estimates without bias correction.

Scripture [77] (archive ScriptureScorer.jar provided by the developers on 6 March 2014) is a tool that was designed for reconstructing and estimating the relative likelihoods of different isoforms. Scripture takes as input a file of transcript annotations (in BED12 format) and a SAM or BAM file with read-to-genome alignments, indexed and sorted by coordinates. We ran Scripture based on instructions provided to us by its developers as follows:

- java -Xmx<XX>g -jar ScriptureScorer.jar \ -annotations \
- <annotations.bed> -alignments \ <alignments_genome.bam> -strand \
- <first> -singleEnd -minMappingQuality \ 5 -out <out_file> \

TIGAR2 [78] (update from 6 March 2014) takes as input a FASTA file of transcript sequences and a SAM or BAM genome alignments file, sorted by read name (randomized). We used the following command to run TIGAR2:

- java -Xms<XX>g -Xmx<XX>g -jar Tigar2.jar \ <sequences.fa> <alignments_genome.bam> \ --alpha_zero 0.1 <out_file> \

### Normalization and stratification of expression ‘ground truths’ and estimates

In order to assess the accuracy of expression level estimates, we first converted the ‘ground truth’ transcript abundances provided in the Flux Simulator output for the simulated data and the by the A-seq-2 data (processed as described above) for the human and mouse samples to a standard library size of 1 million reads. We refer to these measures as transcripts per million transcripts (TPM) and processing regions per million processing regions (PPM), respectively. Since the benchmarked methods already supplied estimates in normalized expression units, no further processing of these values was required. In particular, we have used the reads/fragments per kilobase of exon model per million mapped reads (RPKM/FPKM) units wherever available, thus accounting not only for differences in library sizes but also for differences in transcript lengths. The latter is necessary because the number of fragments obtained from a given RNA during library preparation, and thus the read count for that transcript, is proportional to its length [79]. Only in the case of MMSEQ we have used the exponential of the reported means of the posterior distributions μ instead of RPKM (see above). However, these units are largely equivalent as they both control for sample size and feature length [73]. In cases where estimates were absent for individual transcripts, these were set to zero. For the comparisons of RPKM estimates with A-seq-2-based estimates (human and mouse), only those poly(A)-processing regions were considered that correspond to the ends of transcripts annotated in the GENCODE annotation sets (and vice versa). However, to account for the fact that only poly(A)-containing transcripts are efficiently captured by our sequencing library preparation protocols, we only considered transcripts which we presume could have been polyadenylated (annotated as either ‘antisense’, ‘lincRNA’, ‘nonsense_mediated_decay’, ‘processed_pseudogene’, ‘processed_transcript’, ‘protein_coding’, or ‘retained_intron’). RPKM estimates for the remaining processing sites (25,393 and 17,183 for v19 human assembly version and M2 mouse assembly version, respectively) were then obtained by summing the RKPM values of the transcripts ending at individual poly(A)-processing regions. Similarly, we calculated gene-level expression estimates by summing the RPKM values of all transcripts (simulated data) or the TPM values of all processing regions (human and mouse data) annotated for each gene. Some of the benchmarked methods (Cufflinks, IsoEM, MMSEQ, RSEM, and rSeq) already provide gene-level estimates. However, for Cufflinks and MMSEQ these are not fully equivalent to the sums computed as described above. In the case of Cufflinks, this is apparently because of residual counts that could not be confidently assigned to any of the isoforms of a gene, since in the transcript output for that method (‘isoforms.fpkm_tracking’) there is reported for each gene an estimate that accounts for the difference between the sum of transcript isoform estimates and the gene expression estimates reported in a separate file (‘genes.fpkm_tracking’). For MMSEQ, gene level estimates are produced by aggregating the Markov chain Monte Carlo traces for the transcripts originating from a gene locus. Whenever gene-level estimates of expression were directly reported by a method, we have used these. As with transcripts, missing gene expression estimates were set to zero.

### Count-based gene-level estimates of expression

Although our primary interest was to assess the accuracy of methods for isoform expression profiling, a lot of studies rather limit themselves to gene-level estimates of gene expression. The question then arises of how the methods that are used for obtaining gene-level estimates compare with those that are specifically designed for estimating isoform abundance but can be co-opted for the estimation of gene-level expression levels as well. One method for estimating gene-level expression is ‘union exon’-based counting. To implement this method we have selected the exon entries from the GENCODE annotation files, grouped them by the ENSEMBL gene identifier, and merged overlapping exons for each gene. When analyzing human or mouse data, we have discarded the exons of transcripts that do not end in annotated poly(A)-processing regions or that are unlikely to be polyadenylated, analogous to the filtering that we applied to transcripts used in the benchmarking (see above). We then generated per-gene counts by intersecting the genomic alignments of the different datasets with the resulting ‘pseudoexons’, using the function summarizeOverlaps of the R/Bioconductor package GenomicAlignments [80] with options --ignoreStrand=FALSE, --mode=‘IntersectionStrict’ and --interFeature=TRUE. While this procedure prevents double-counting of reads and is frequently applied in the context of gene counting in RNA-seq experiments, reads aligning to multiple genomic loci as well as those aligning to loci for which more than one feature is annotated are not considered. Additionally, many read alignments covering exon-exon-junctions are discarded because these exon-exon junctions are not part of the set of junctions between pseudo-exons. To appropriately handle such cases we implemented also a ‘transcript’-based counting method as follows: We used the R/Bioconductor package rtracklayer [66] to convert the GENCODE-annotated exons of either all (*in silico*-generated data) or the filtered set of transcripts (human and mouse data; see above) to the BED12 / BED12+3 format, a tabular format able to encode gaps. We then intersected the genomic alignments for each dataset with the corresponding annotation file using bedtools mode intersect [81] such that overlaps were only reported if the entire read alignments, including the gaps that could correspond to introns, matched the transcript alignments on the sense strand (options -s and -f 1). The resulting overlaps were summarized, further distributing reads equally to all (possibly overlapping) annotated loci to which they aligned with the same edit distance. Thus, we first determined the number of genomic loci *l* for which overlaps were reported for a given read. For each of these, we then added $\frac{1}{\varSigma_{i=1..l}{g}_i}$ to the total count of all genes that give rise to one or more transcripts from a locus *i*. For each library, the counts produced by each of these counting methods were then converted to RPKM by dividing by (1) the total number of reads that could be successfully aligned to the genome and (2) the total length (in nucleotides) of the ‘union exons’ (see above) of the considered transcripts, followed by multiplication by 1 billion.

### Evaluating the accuracy of gene/isoform abundance estimates

We assessed the accuracy of the methods in terms of Spearman correlation coefficients between the known (simulated data) or independently estimated abundances (A-seq-2) and the abundances inferred with the individual methods. Depending on the type of data and analysis, we applied this procedure on the level of transcripts, poly(A)-processing regions, and/or genes, either considering all features or subsets thereof, grouped by common features (for example, expression ranges, structural). Where indicated, we have further computed the Pearson correlation coefficient and the root mean square error. In these cases, we have first set all expression levels (true or estimated) below 0.03125 (the log2 of which is −5) to that value and log2-transformed the resulting ‘pseudocount’-adjusted values.

### Availability of supporting data

Raw sequencing (RNA-seq and A-seq-2) and *in silico*-generated read files are available in the Sequence Read Archive (SRA) [82] repository under accession SRP051039 [83]. As the SRA currently only supports the deposition of read alignments to genomic sequences, we have hosted the processed transcriptome alignment files, corresponding to the simulated/synthetic and experimental (RNA-seq) read libraries, on our companion website [84]. The page further includes information on where to find the benchmarked methods, all source code - organized in well documented convenient wrappers that allow easy recreation of either the whole study or parts thereof - and an online analysis service where users can upload expression estimates inferred from the datasets used in this study and compare them to the methods (or their specific versions) assessed here.

## Additional files

Additional file 1: Figure S1. Overview of the study design. Sequencing data (blue boxes; 1) were generated synthetically (Flux Simulator; left side) or experimentally (right side) from human or mouse cells, following either a regular RNA-seq (blue arrows) or an A-seq-2 3′ end sequencing protocol (red arrows). 3′ adapters (if present) and poly(A)-tails were removed from read sequences (‘pre-processing’), and the trimmed reads were then aligned against both the genome and the transcriptome (green boxes; 2). Genome alignments were supplemented with read alignments covering splice junctions by converting transcriptome alignments to genome coordinates. Genome and transcriptome alignments were then compared to ensure that only the best alignments were kept for each read. Based on the remaining alignments (genome or transcriptome, depending on requirements), expression estimates were computed (red boxes) either with the surveyed, model-based methods (3a), or count-based methods (RNA-seq: 3b, A-seq-2: 3c). Subsequently (‘post-processing’), the raw numbers produced by the latter methods, as well as the true number of expressed transcripts in the synthetic dataset (as provided by Flux Simulator; gray arrow), were normalized, and the normalized expression estimates were extracted from the outputs of the surveyed model-based inference methods. Depending on the downstream analysis, expression estimates for transcripts and 3′ end processing sites (‘Poly(A)’) were aggregated and filtered (purple boxes; 4). To evaluate the performance of the surveyed methods (magenta boxes; 5), the accuracy of the surveyed transcripts abundance inference methods were analyzed by comparing the produced estimates to either the ground truth expression (synthetic data) or the A-seq-2-based estimates (experimental data). Additionally, runtime and memory consumption was evaluated. Steps at which either transcript/gene annotations (GENCODE) or transcript sequences (ENSEMBL) were used are marked with white triangles at the upper left corners. Refer to the Methods section and the main text for further details. Additional file 2: Figure S2. Multithreading efficiency and running time / memory footprint trade-off. Transcript isoform abundances were estimated with each of the indicated methods based on *in silico*-generated sequencing datasets. (A) The efficiency of multi-core use is indicated in terms of the speedup factor (ratio of running times when using 1 compared to 16 cores) for different sequencing depths. (B and C) Relationships between running time and memory footprint when processing 100 million reads with either 1 (B) or 16 (C) cores. Note that data for TIGAR2 are unavailable for (A) and (C), because the method does not support the use of multiple cores. Additional file 3: Figure. S3. Accuracy of transcript isoform abundance estimates inferred from *in silico*-generated sequencing data. For each method, correlations between true and inferred transcript abundances are shown as heat density plots. The corresponding Spearman correlation coefficients (r_s_) are indicated. Estimates were produced based on the 30 million read dataset. (A) BitSeq. (B) CEM. (C) Cufflinks. (D) eXpress. (E) IsoEM. (F) MMSEQ. (G) RSEM. (H) rSeq. (I) Sailfish. (J) Scripture. (K) TIGAR2. Additional file 4: Figure S4. Comparison of different metrics for quantifying the accuracy of isoform abundance estimates. The accuracy of expression level estimates with respect to the ground truth was assessed by the Spearman and Pearson correlation coefficients, as well as the root mean square error (RMSE). The values obtained for expressed transcripts (A) and expressed genes (B) are plotted. Color intensities have been computed per column by scaling raw values such that the best value (high for correlation coefficients, low for RMSE) corresponds to the most intense and the worst to the least intense color. Additional file 5: Figure S5. Accuracy of gene expression estimates inferred from *in silico*-generated sequencing data. As in Additional file 3: Fig. S3, but estimates were produced for genes instead of transcripts. (A) BitSeq. (B) CEM. (C) Cufflinks. (D) eXpress. (E) IsoEM. (F) MMSEQ. (G) RSEM. (H) rSeq. (I) Sailfish. (J) Scripture. (K) TIGAR2. (L) Counting method ‘transcript’. (M) Counting method ‘union exon’. Additional file 6: Figure S6. Accuracy of ‘present calls’. The ability of each method to accurately determine whether a given transcript or gene is expressed was determined by calculating false positive (A and B) and true positive (C through F) rates across different sequencing depths. A transcript (A, C, and E) or gene (B, D, and F) was considered expressed, if it has - according to the ground truth - a non-zero expression. In contrast to A through D, where all features are considered, panels E and F show the true positive rates only for lowly expressed transcripts and genes (log2 TPM <0 and <1.1, respectively; compare expression bins in Fig. 2). Note that by default, BitSeq and MMSEQ report small non-zero ‘priors’. For these methods, we included modified estimates (‘priors’ to 0), in which a portion of these small values were set to zero according to simple algorithms (refer to the main text and the Methods section for details). Additional file 7: Figure S7. Accuracy of expression estimates across all transcripts and genes. As in Fig. 2a and b, but including, respectively, transcripts (A) and genes (B) that are not expressed according to the ground truth. Additional file 8: Figure S8. Effect of ‘native’ short-read aligners. For methods strongly recommending the use of a specific short-read aligner (CEM, Cufflinks, MMSEQ, Scripture) or using such an aligner internally by default (RSEM), expression levels inferred based on alignments obtained with the respective aligners were compared to the estimates produced following our own processing and alignment pipeline. Accuracies were calculated across different read depths as in Fig. 2, either for expressed transcripts (A) or genes (B), or for all transcripts (C) or genes (D). Additional file 9: Figure S9. Impact of bias correction settings on simulated data. For methods where an optional sequencing/positional bias correction setting is implemented, we have compared estimation accuracies obtained when executing the programs with the respective options set or unset. Accuracies were calculated for 30 million reads as in Fig. 2, either for transcripts (A) or genes (B). Default settings (that were also used throughout this study if not indicated otherwise) are indicated in parentheses after the method name (circle: bias correction off, triangle: bias correction on). Note that Cufflinks also has a bias correction option (--frag-bias-correct; default: off). However, in our hands the program crashed when this option was specified. Additional file 10: Figure S10. Expression level distributions across bins of transcripts and genes. All transcripts or genes expressed at levels of 0 < log2 TPM <5.5 were distributed across bins according to transcript length (A), GC content (B), the number exons per transcript (C), and the number of transcripts per gene (D). Ranges of the corresponding values covered by each bin are indicated in the legends to each chart, together with the number of features (transcripts or genes) they contain. The expression level distributions of the features in each bin are depicted as cumulative distribution functions. Additional file 11: Figure S11. Cufflinks-based abundance estimates of single-exon transcripts. Cufflinks was used to infer transcript isoform expression levels from the alignments of 30 million *in silico*-generated reads. Alignments were produced either following our own segemehl-based pipeline (A to C) or by TopHat (D to F). Estimated abundances are plotted against true abundances for transcripts expressed at 0 < log2 TPM <5.5 and comprising either one exon (A and D), two exons (B and E), or 11 or more exons (C and F). Heat map colors reflect the densities of data points and the corresponding Spearman correlation coefficients (r_s_) are indicated. For all single-exon transcripts expressed at 0 < log2 TPM <5.5, transcript isoform abundances as estimated by Cufflinks are plotted against true abundances. Additional file 12: Figure S12. Impact of gene structural features on expression estimates. Transcripts and genes have been distributed over different bins according to the indicated structural features (see Fig. 3 and main text). The variation between estimation accuracies for these bins are indicated in terms of the standard deviations σ of the Spearman correlation coefficients between ground truth and estimates. Additional file 13:
**List of the** ***P*** **values of the Kolmogorov-Smirnov goodness of fit tests for the assessment of the impact of structural properties (Fig.** 3 **) and gene biotypes (Fig.** 5c, d **) on estimation accuracy.** Tests were performed on the log-ratios of estimated versus ‘ground truth’ (simulated or A-seq-2) expression for a given subset of transcripts/genes and for the whole set of transcripts/genes used for a given analysis (for structural properties and synthetic data the whole set was composed of those transcripts expressed at 0 < log2 TPM <5.5 and those genes expressed at 0 < log2 TPM <6.2; for gene biotypes, human and mouse data: the full set contained all genes with 3′ end processing sites for which read evidence was found in the analysis of our A-seq-2 data). Additional file 14: Figure S13. Agreement between expression level estimates for replicates of NIH/3T3 cells. Transcript isoform and gene abundances were estimated with each of the indicated methods based on RNA-seq data obtained from two biological replicates of murine NIH/3T3 cells. (A) The agreement between expression estimates for the two replicates are indicated as Spearman correlation coefficients r_s_, both at the level of transcripts and genes. (B) A-seq-2-based 3′ end processing site expression level estimates for the two replicates are plotted against each other. The Spearman correlation coefficient r_s_ is indicated. (C) As in (B), but estimates are compared at the level of gene expression. (D) As in (A), but with the addition of 3′ end processing site abundances. For computing expression estimates for either feature type (transcript, 3′ end processing site, and gene), only those transcripts are considered that end in annotated 3′ end processing sites (see main text and Methods for details). Additional file 15: Figure S14. Replicate agreement between abundance estimates for features corresponding to expressed 3′ end processing sites. As in Figs. 4d and Additional file 14: Fig. S13D, but with the further requirement that the considered transcripts need to end in annotated 3′ end processing sites that show evidence of expression, according to the A-seq-2 analysis. Results are shown for replicates of (A) human Jurkat cells and (B) murine NIH/3T3 cells. Additional file 16: Figure S15. Accuracy of 3′ end processing site abundance estimates inferred from Jurkat sequencing data. Transcript abundances inferred by the surveyed methods from RNA-seq libraries prepared from human Jurkat cells (replicate 1) were aggregated by 3′ end processing sites and plotted against the corresponding estimates obtained by the analysis of A-seq-2 sequencing data. Heat map colors represent data point densities and Spearman correlation coefficients (r_s_) are indicated. (A) BitSeq. (B) CEM. (C) Cufflinks. (D) eXpress. (E) IsoEM. (F) MMSEQ. (G) RSEM. (H) rSeq. (I) Sailfish. (J) Scripture. (K) TIGAR2. Additional file 17: Figure S16. Accuracy of 3′ end processing site abundance estimates inferred from NIH/3T3 sequencing data. As in Additional file 16: Fig. S15, but data were from murine NIH/3T3 cells. Additional file 18: Figure S17. Impact of bias correction settings on abundance estimates from experimental data. As in Additional file 9: Fig. S9, but expression estimates were obtained for human (A and B) or mouse (C and D) cells and also include estimation accuracies on the level of 3′ end processing sites. Spearman correlation coefficients were calculated by comparison to A-seq-2 estimates (see Fig. 5) rather than the simulation ground truth. (A) Jurkat data, replicate 1. (B) Jurkat data, replicate 2. (C) NIH/3T3 data, replicate 1. (D) NIH/3T3 data, replicate 2. Additional file 19:
**Parameter file in CSV format for use with the Flux Simulator software [** 26 **].**

## Abbreviations

PPM: processing regions per million processing regions
RPKM/FPKM: reads/fragments per kilobase of exon model per million mapped reads
TPM: transcripts per million transcripts
