# Supplementary figures and images for: Comparative assessment of methods for the computational inference of transcript isoform abundance from RNA-seq data
Source: Genome Biol. 2015 Jul 23;16(1):150. doi: 10.1186/s13059-015-0702-5 (PMC4511015; doi:10.1186/s13059-015-0702-5)

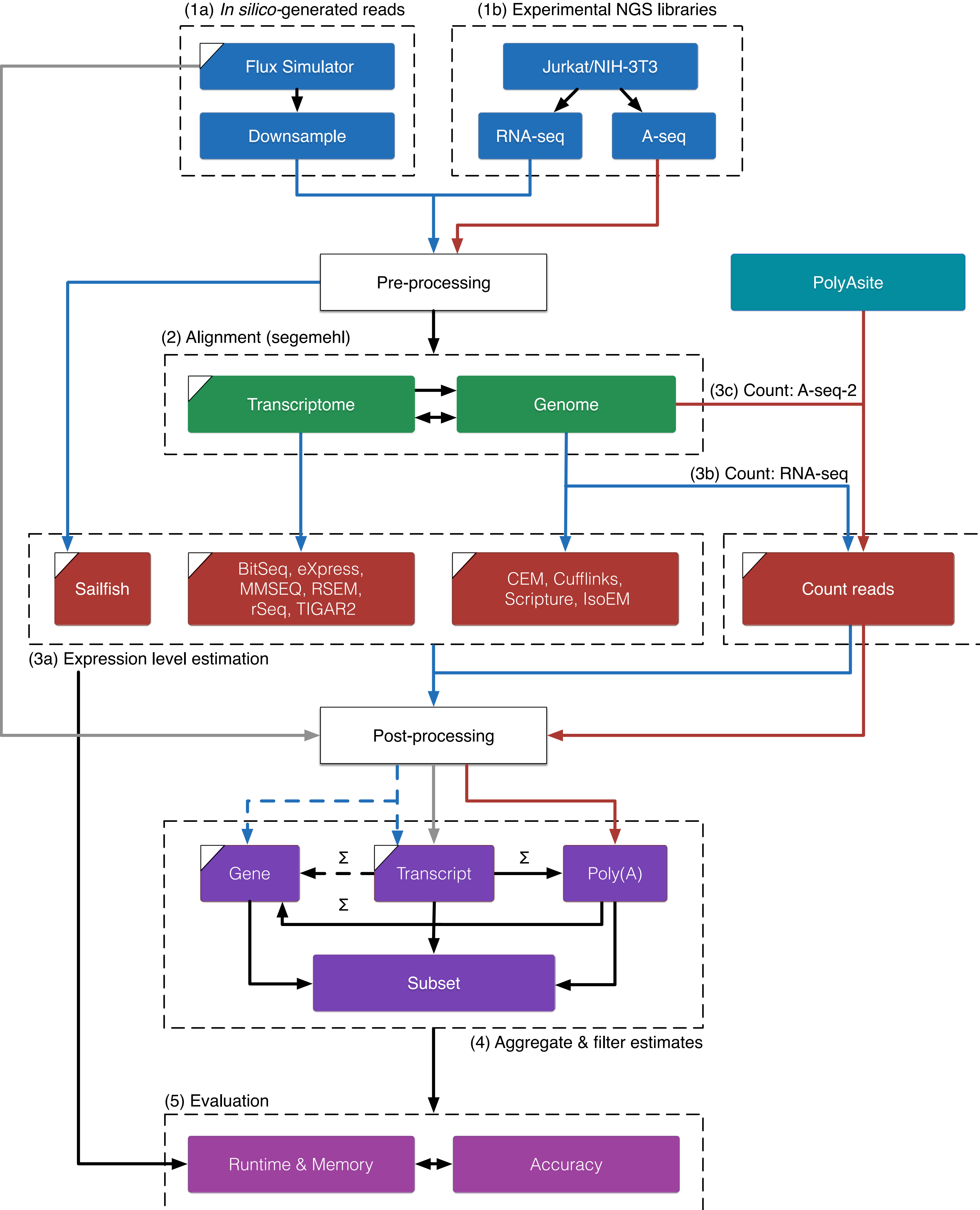

Supplement: Additional file 1: Figure S1. — Overview of the study design. Sequencing data (blue boxes; 1) were generated synthetically (Flux Simulator; left side) or experimentally (right side) from human or mouse cells, following either a regular RNA-seq (blue arrows) or an A-seq-2 3′ end sequencing protocol (red arrows). 3′ adapters (if present) and poly(A)-tails were removed from read sequences (‘pre-processing’), and the trimmed reads were then aligned against both the genome and the transcriptome (green boxes; 2). Genome alignments were supplemented with read alignments covering splice junctions by converting transcriptome alignments to genome coordinates. Genome and transcriptome alignments were then compared to ensure that only the best alignments were kept for each read. Based on the remaining alignments (genome or transcriptome, depending on requirements), expression estimates were computed (red boxes) either with the surveyed, model-based methods (3a), or count-based methods (RNA-seq: 3b, A-seq-2: 3c). Subsequently (‘post-processing’), the raw numbers produced by the latter methods, as well as the true number of expressed transcripts in the synthetic dataset (as provided by Flux Simulator; gray arrow), were normalized, and the normalized expression estimates were extracted from the outputs of the surveyed model-based inference methods. Depending on the downstream analysis, expression estimates for transcripts and 3′ end processing sites (‘Poly(A)’) were aggregated and filtered (purple boxes; 4). To evaluate the performance of the surveyed methods (magenta boxes; 5), the accuracy of the surveyed transcripts abundance inference methods were analyzed by comparing the produced estimates to either the ground truth expression (synthetic data) or the A-seq-2-based estimates (experimental data). Additionally, runtime and memory consumption was evaluated. Steps at which either transcript/gene annotations (GENCODE) or transcript sequences (ENSEMBL) were used are marked with white triangles at the upper left c [file 13059_2015_702_MOESM1_ESM.pdf]

A

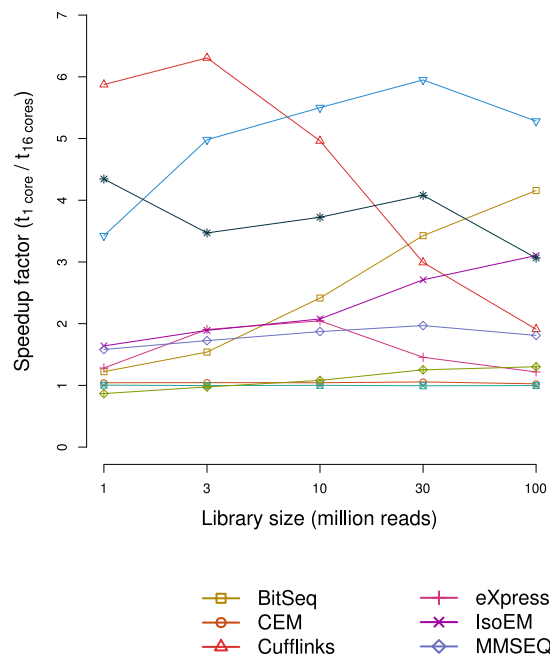

B

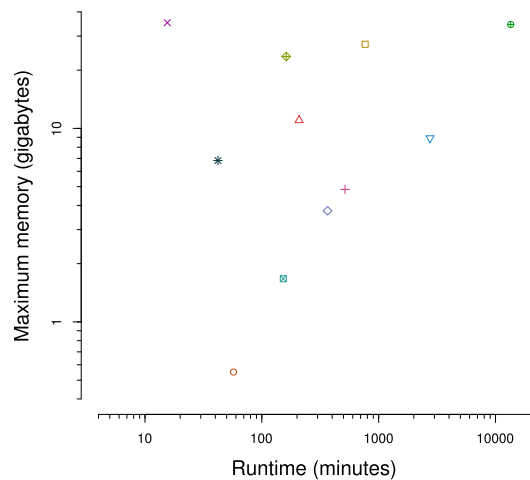

C

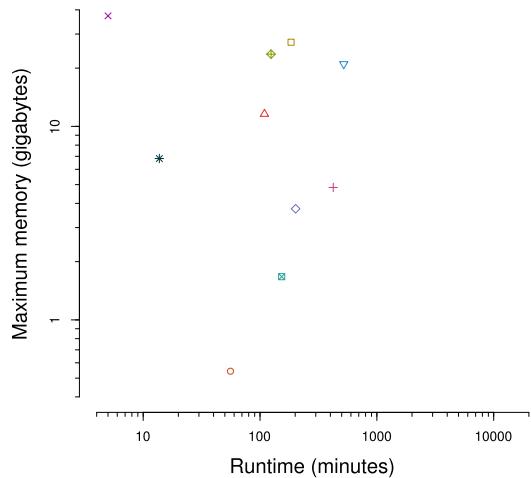

Supplement: Additional file 2: Figure S2. — Multithreading efficiency and running time / memory footprint trade-off. Transcript isoform abundances were estimated with each of the indicated methods based on in silico-generated sequencing datasets. (A) The efficiency of multi-core use is indicated in terms of the speedup factor (ratio of running times when using 1 compared to 16 cores) for different sequencing depths. (B and C) Relationships between running time and memory footprint when processing 100 million reads with either 1 (B) or 16 (C) cores. Note that data for TIGAR2 are unavailable for (A) and (C), because the method does not support the use of multiple cores. [file 13059_2015_702_MOESM2_ESM.pdf]

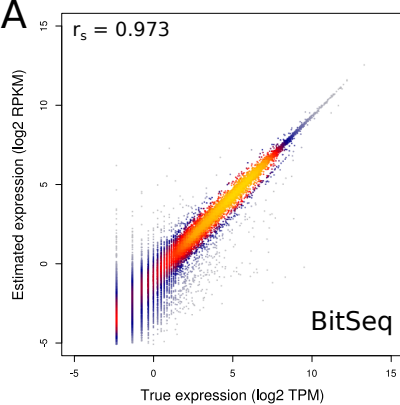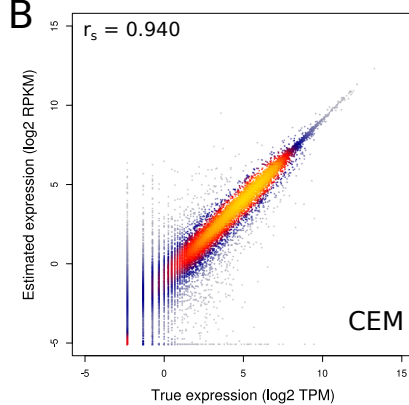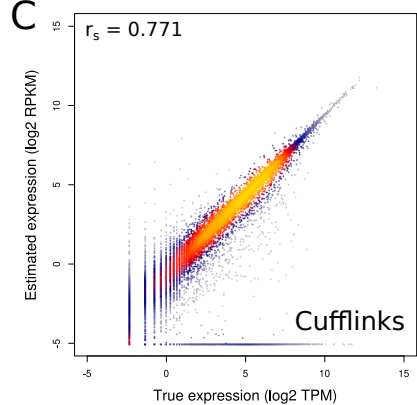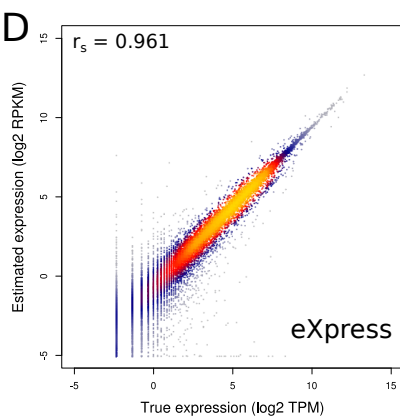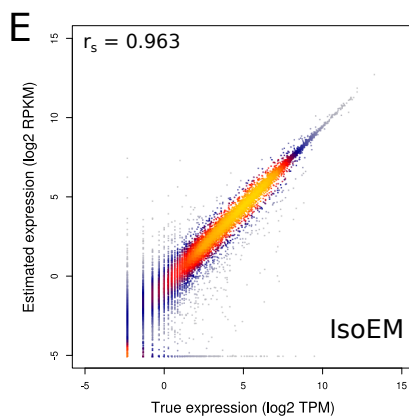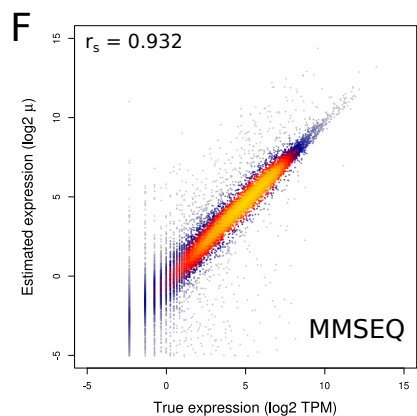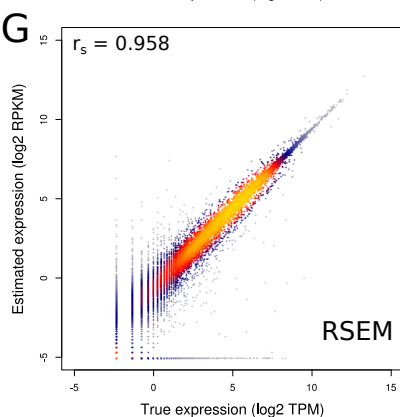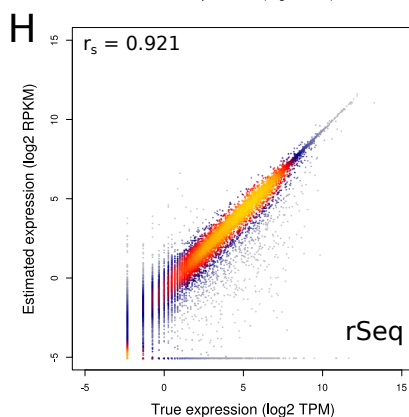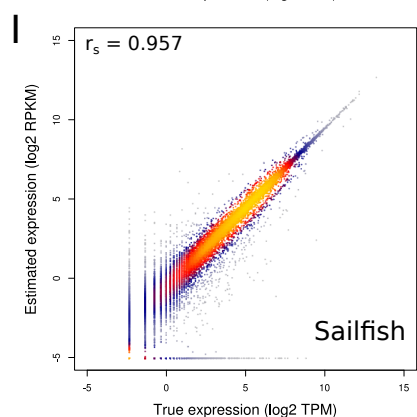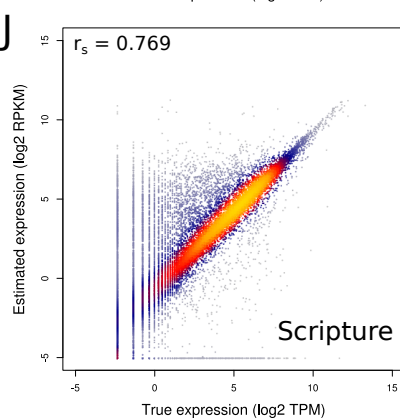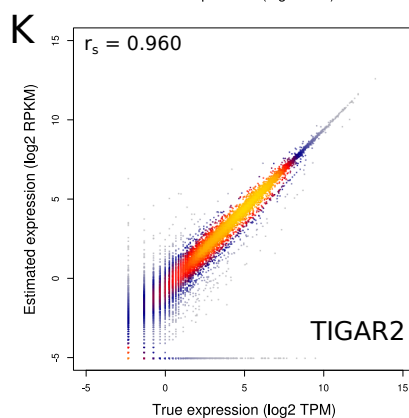

Supplement: Additional file 3: Figure. S3. — Accuracy of transcript isoform abundance estimates inferred from in silico-generated sequencing data. For each method, correlations between true and inferred transcript abundances are shown as heat density plots. The corresponding Spearman correlation coefficients (rs) are indicated. Estimates were produced based on the 30 million read dataset. (A) BitSeq. (B) CEM. (C) Cufflinks. (D) eXpress. (E) IsoEM. (F) MMSEQ. (G) RSEM. (H) rSeq. (I) Sailfish. (J) Scripture. (K) TIGAR2. [file 13059_2015_702_MOESM3_ESM.pdf]

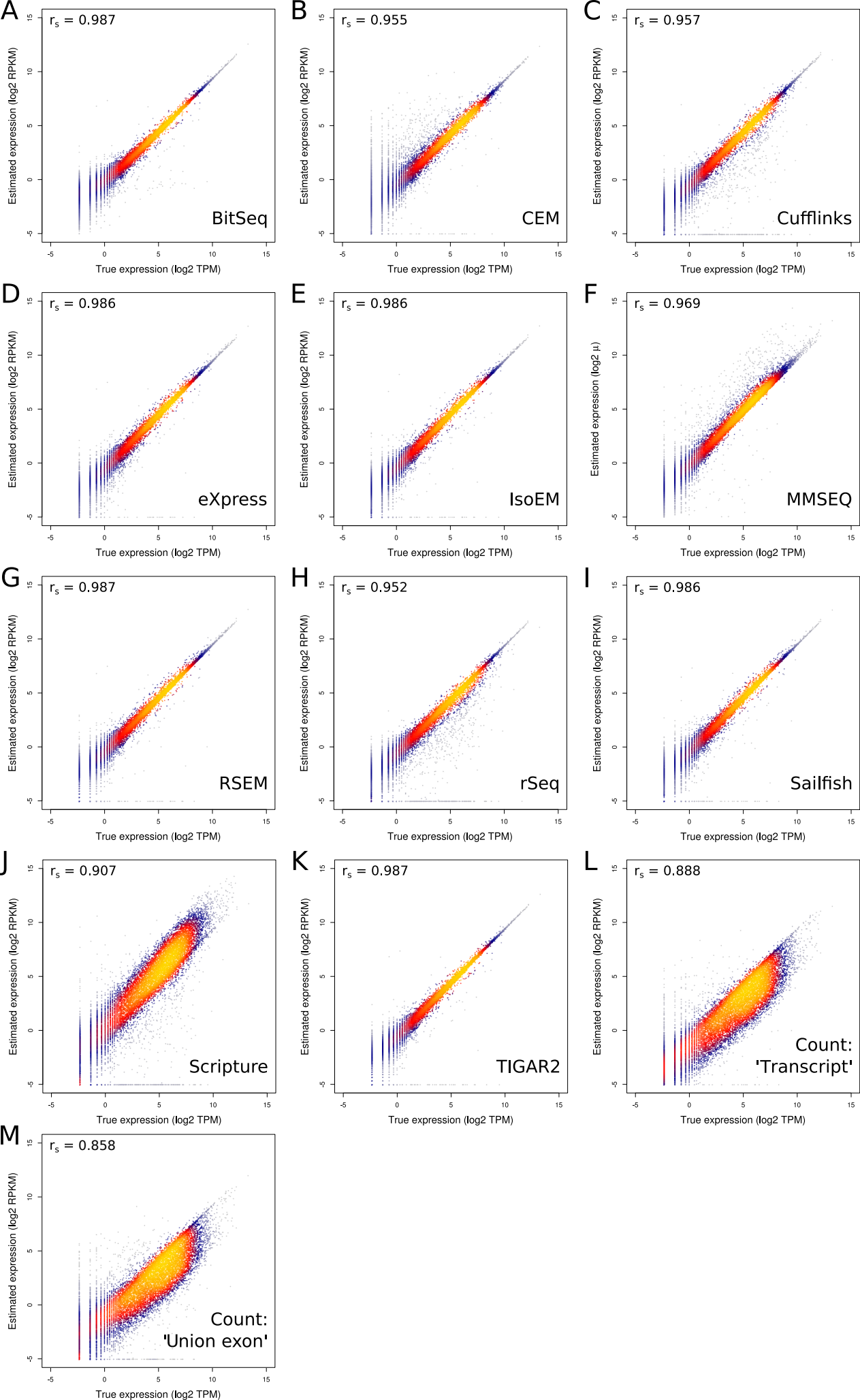

Supplement: Additional file 5: Figure S5. — Accuracy of gene expression estimates inferred from in silico-generated sequencing data. As in Additional file 3: Fig. S3, but estimates were produced for genes instead of transcripts. (A) BitSeq. (B) CEM. (C) Cufflinks. (D) eXpress. (E) IsoEM. (F) MMSEQ. (G) RSEM. (H) rSeq. (I) Sailfish. (J) Scripture. (K) TIGAR2. (L) Counting method ‘transcript’. (M) Counting method ‘union exon’. [file 13059_2015_702_MOESM5_ESM.pdf]

A

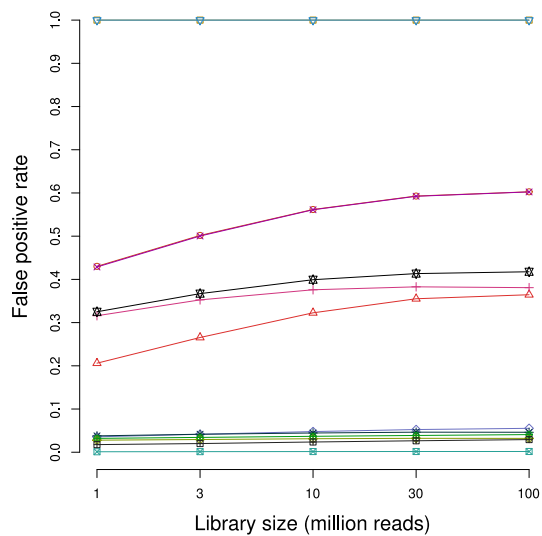

B

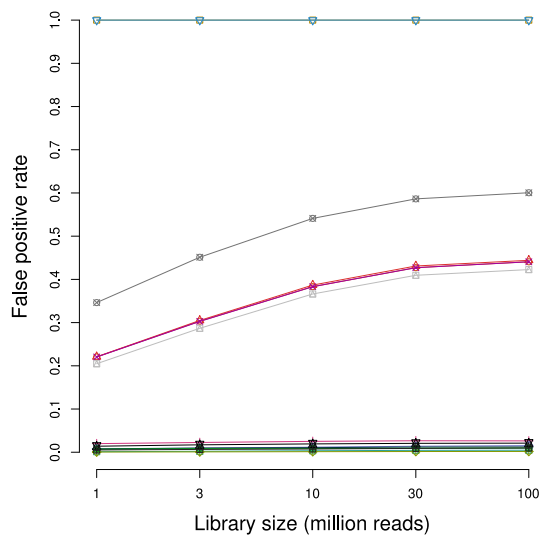

C

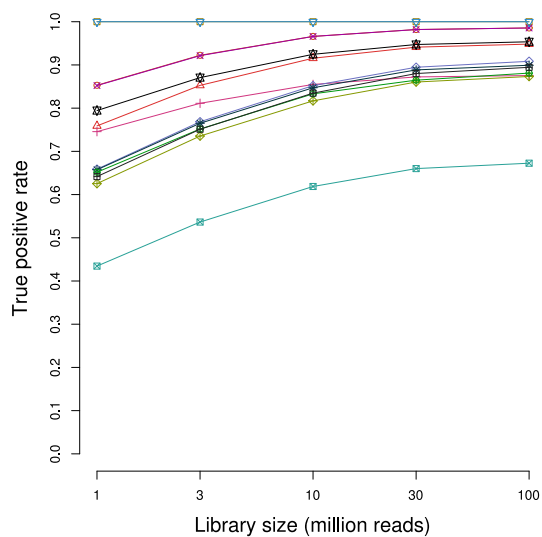

D

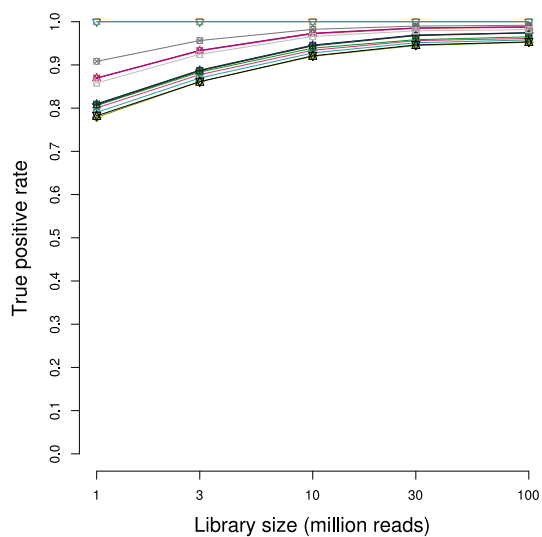

E

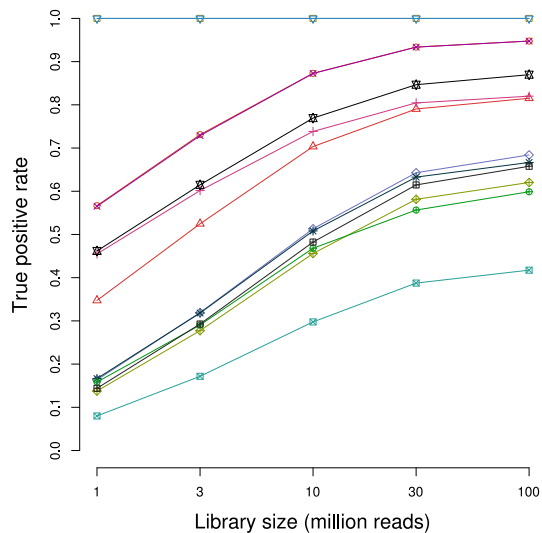

F

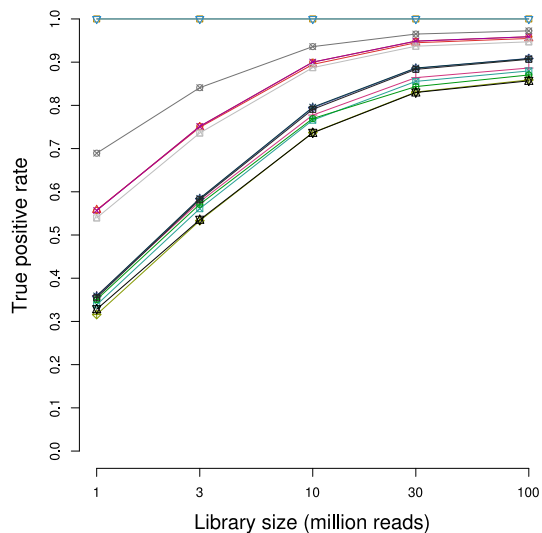

Supplement: Additional file 6: Figure S6. — Accuracy of ‘present calls’. The ability of each method to accurately determine whether a given transcript or gene is expressed was determined by calculating false positive (A and B) and true positive (C through F) rates across different sequencing depths. A transcript (A, C, and E) or gene (B, D, and F) was considered expressed, if it has - according to the ground truth - a non-zero expression. In contrast to A through D, where all features are considered, panels E and F show the true positive rates only for lowly expressed transcripts and genes (log2 TPM <0 and <1.1, respectively; compare expression bins in Fig. 2). Note that by default, BitSeq and MMSEQ report small non-zero ‘priors’. For these methods, we included modified estimates (‘priors’ to 0), in which a portion of these small values were set to zero according to simple algorithms (refer to the main text and the Methods section for details). [file 13059_2015_702_MOESM6_ESM.pdf]

A

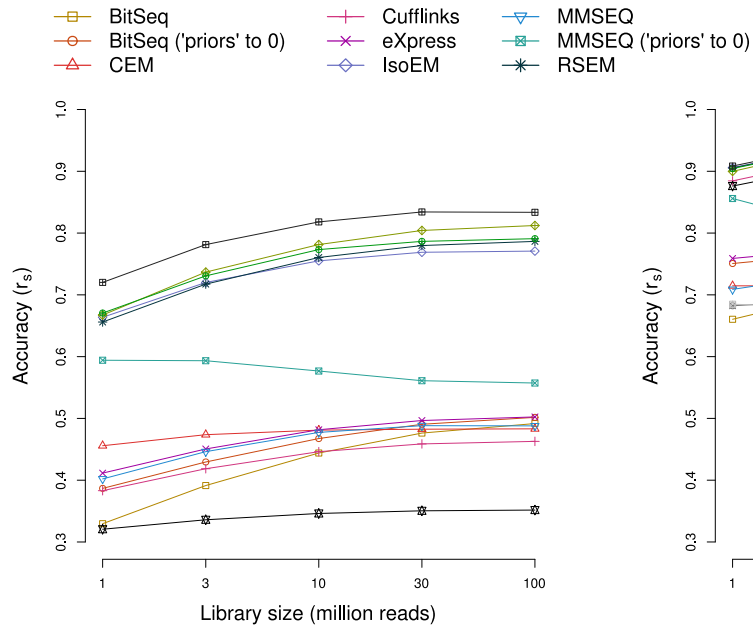

B

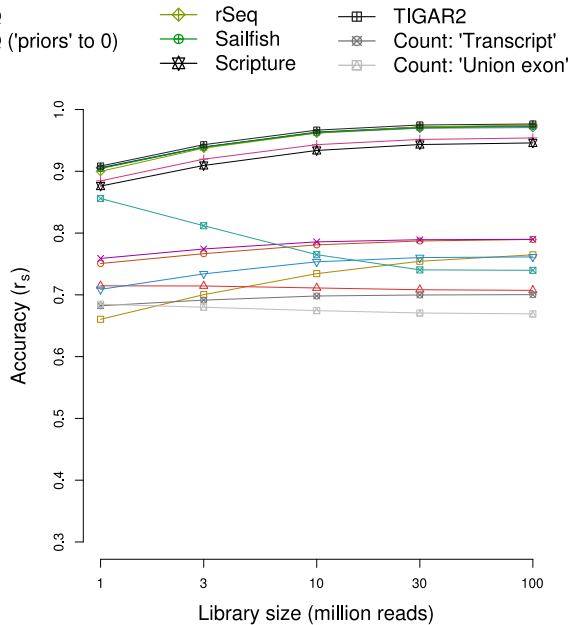

Supplement: Additional file 7: Figure S7. — Accuracy of expression estimates across all transcripts and genes. As in Fig. 2a and b, but including, respectively, transcripts (A) and genes (B) that are not expressed according to the ground truth. [file 13059_2015_702_MOESM7_ESM.pdf]

**A**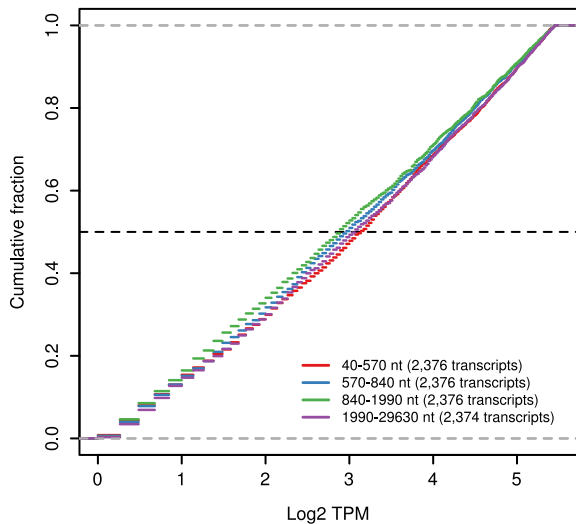**B**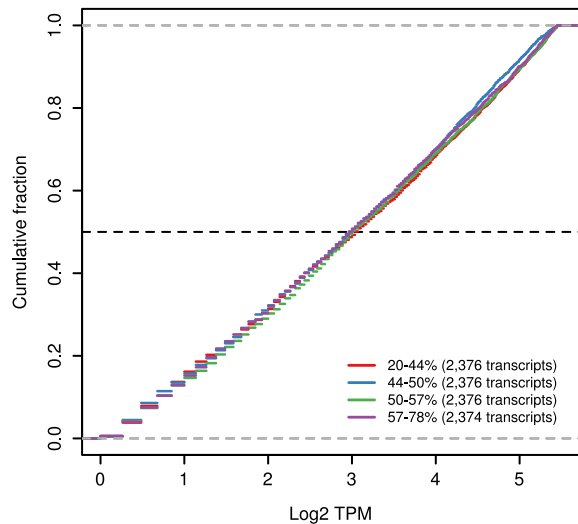**C**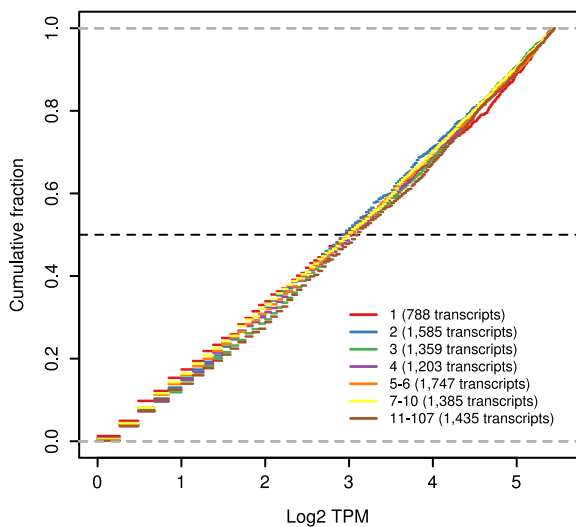**D**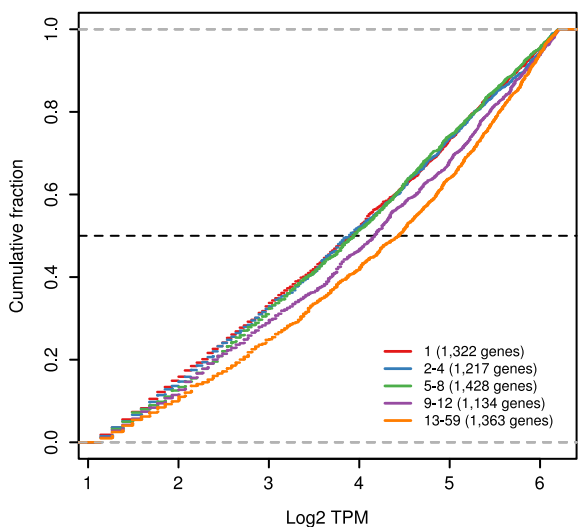

Supplement: Additional file 10: Figure S10. — Expression level distributions across bins of transcripts and genes. All transcripts or genes expressed at levels of 0 < log2 TPM <5.5 were distributed across bins according to transcript length (A), GC content (B), the number exons per transcript (C), and the number of transcripts per gene (D). Ranges of the corresponding values covered by each bin are indicated in the legends to each chart, together with the number of features (transcripts or genes) they contain. The expression level distributions of the features in each bin are depicted as cumulative distribution functions. [file 13059_2015_702_MOESM10_ESM.pdf]

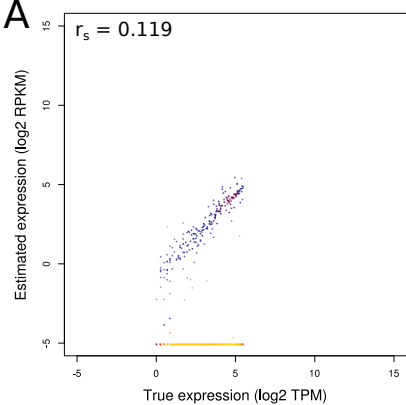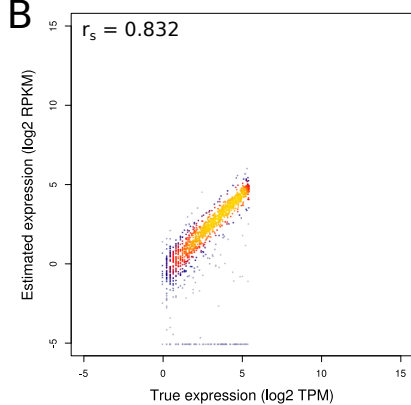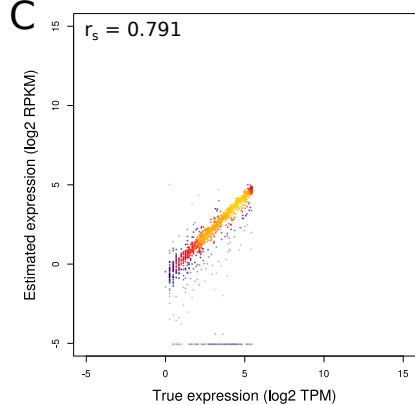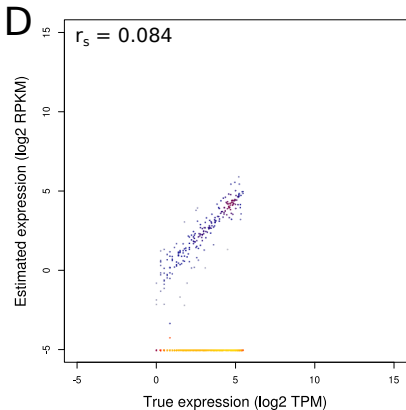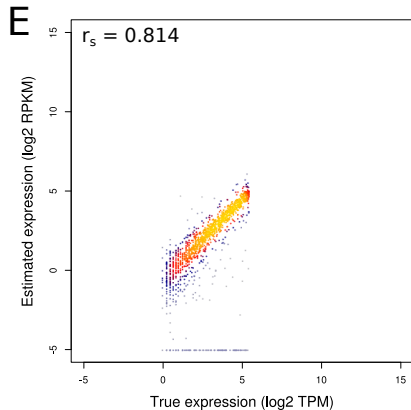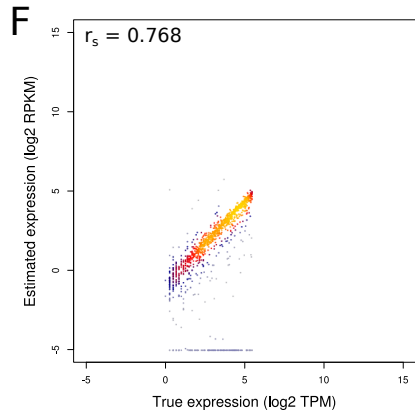

Supplement: Additional file 11: Figure S11. — Cufflinks-based abundance estimates of single-exon transcripts. Cufflinks was used to infer transcript isoform expression levels from the alignments of 30 million in silico-generated reads. Alignments were produced either following our own segemehl-based pipeline (A to C) or by TopHat (D to F). Estimated abundances are plotted against true abundances for transcripts expressed at 0 < log2 TPM <5.5 and comprising either one exon (A and D), two exons (B and E), or 11 or more exons (C and F). Heat map colors reflect the densities of data points and the corresponding Spearman correlation coefficients (rs) are indicated. For all single-exon transcripts expressed at 0 < log2 TPM <5.5, transcript isoform abundances as estimated by Cufflinks are plotted against true abundances. [file 13059_2015_702_MOESM11_ESM.pdf]

□ Transcript length

○ GC content

△ Exons per transcript

◇ Transcripts per gene

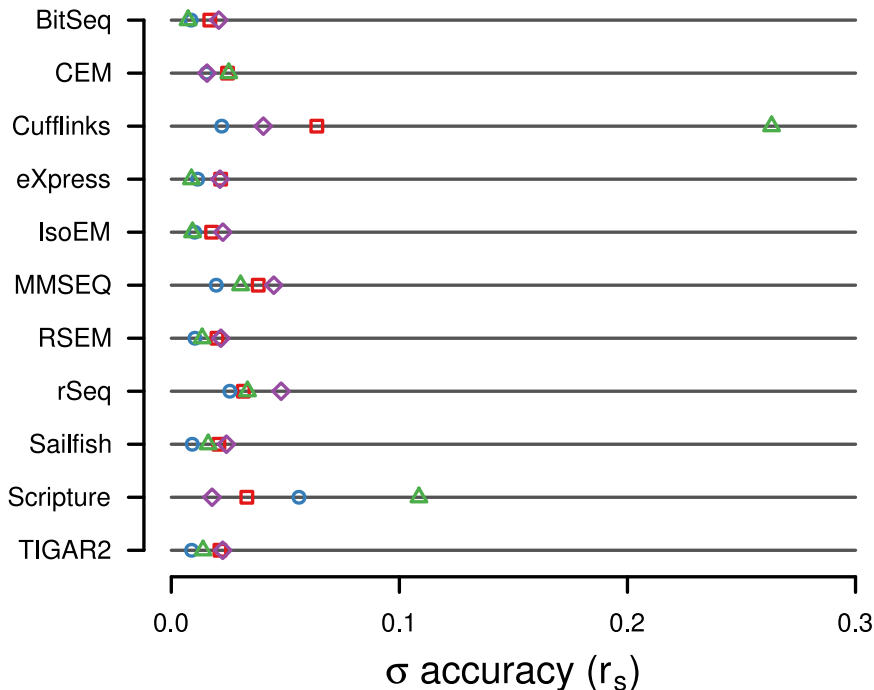

Supplement: Additional file 12: Figure S12. — Impact of gene structural features on expression estimates. Transcripts and genes have been distributed over different bins according to the indicated structural features (see Fig. 3 and main text). The variation between estimation accuracies for these bins are indicated in terms of the standard deviations σ of the Spearman correlation coefficients between ground truth and estimates. [file 13059_2015_702_MOESM12_ESM.pdf]

A

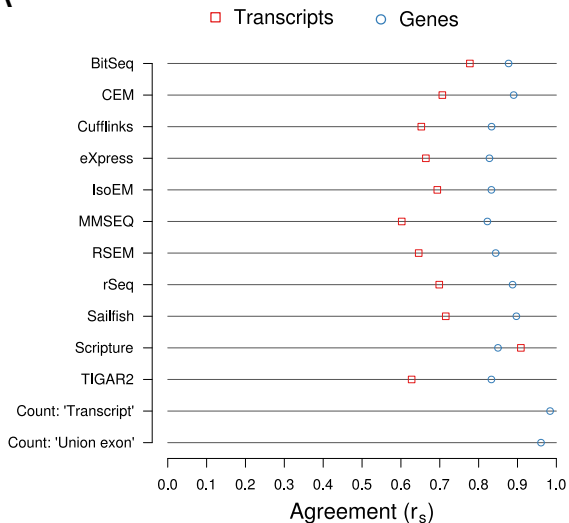

B

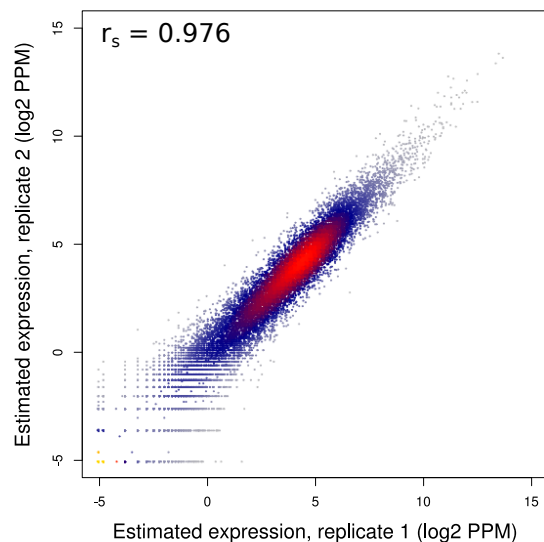

C

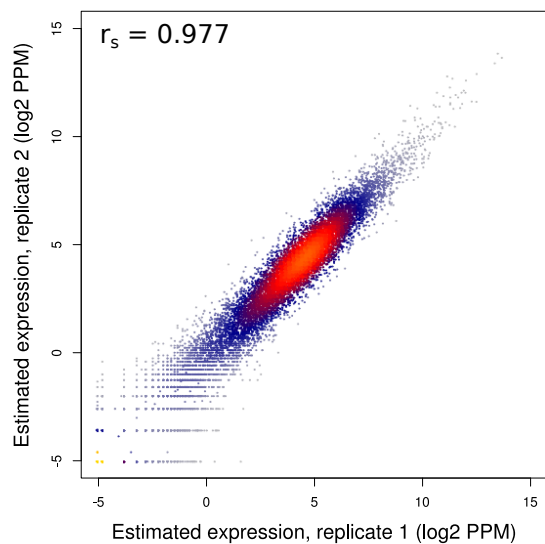

D

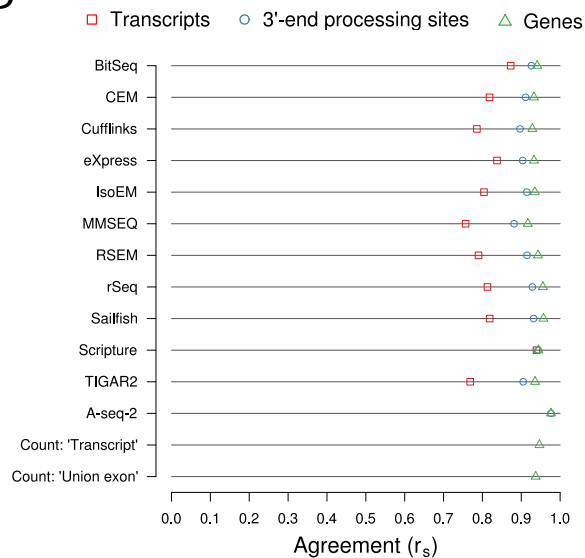

Supplement: Additional file 14: Figure S13. — Agreement between expression level estimates for replicates of NIH/3T3 cells. Transcript isoform and gene abundances were estimated with each of the indicated methods based on RNA-seq data obtained from two biological replicates of murine NIH/3T3 cells. (A) The agreement between expression estimates for the two replicates are indicated as Spearman correlation coefficients rs, both at the level of transcripts and genes. (B) A-seq-2-based 3′ end processing site expression level estimates for the two replicates are plotted against each other. The Spearman correlation coefficient rs is indicated. (C) As in (B), but estimates are compared at the level of gene expression. (D) As in (A), but with the addition of 3′ end processing site abundances. For computing expression estimates for either feature type (transcript, 3′ end processing site, and gene), only those transcripts are considered that end in annotated 3′ end processing sites (see main text and Methods for details). [file 13059_2015_702_MOESM14_ESM.pdf]

A

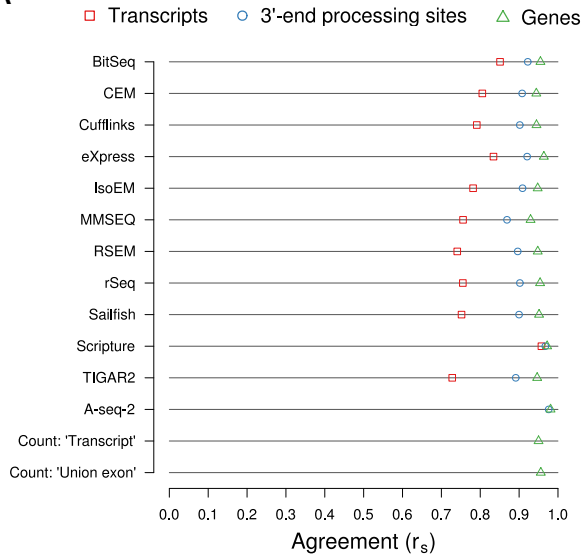

B

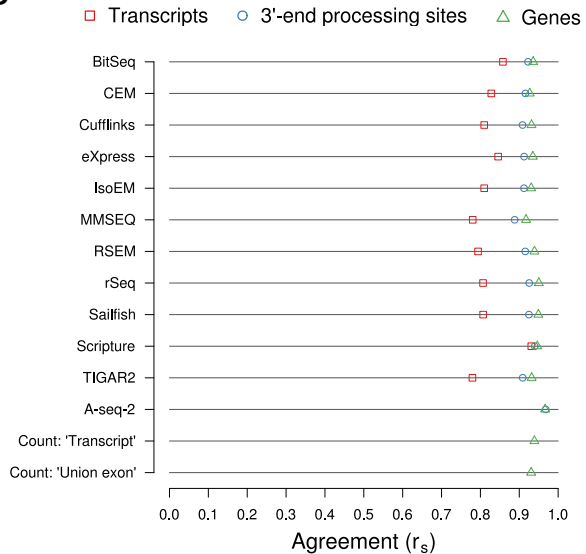

Supplement: Additional file 15: Figure S14. — Replicate agreement between abundance estimates for features corresponding to expressed 3′ end processing sites. As in Figs. 4d and Additional file 14: Fig. S13D, but with the further requirement that the considered transcripts need to end in annotated 3′ end processing sites that show evidence of expression, according to the A-seq-2 analysis. Results are shown for replicates of (A) human Jurkat cells and (B) murine NIH/3T3 cells. [file 13059_2015_702_MOESM15_ESM.pdf]

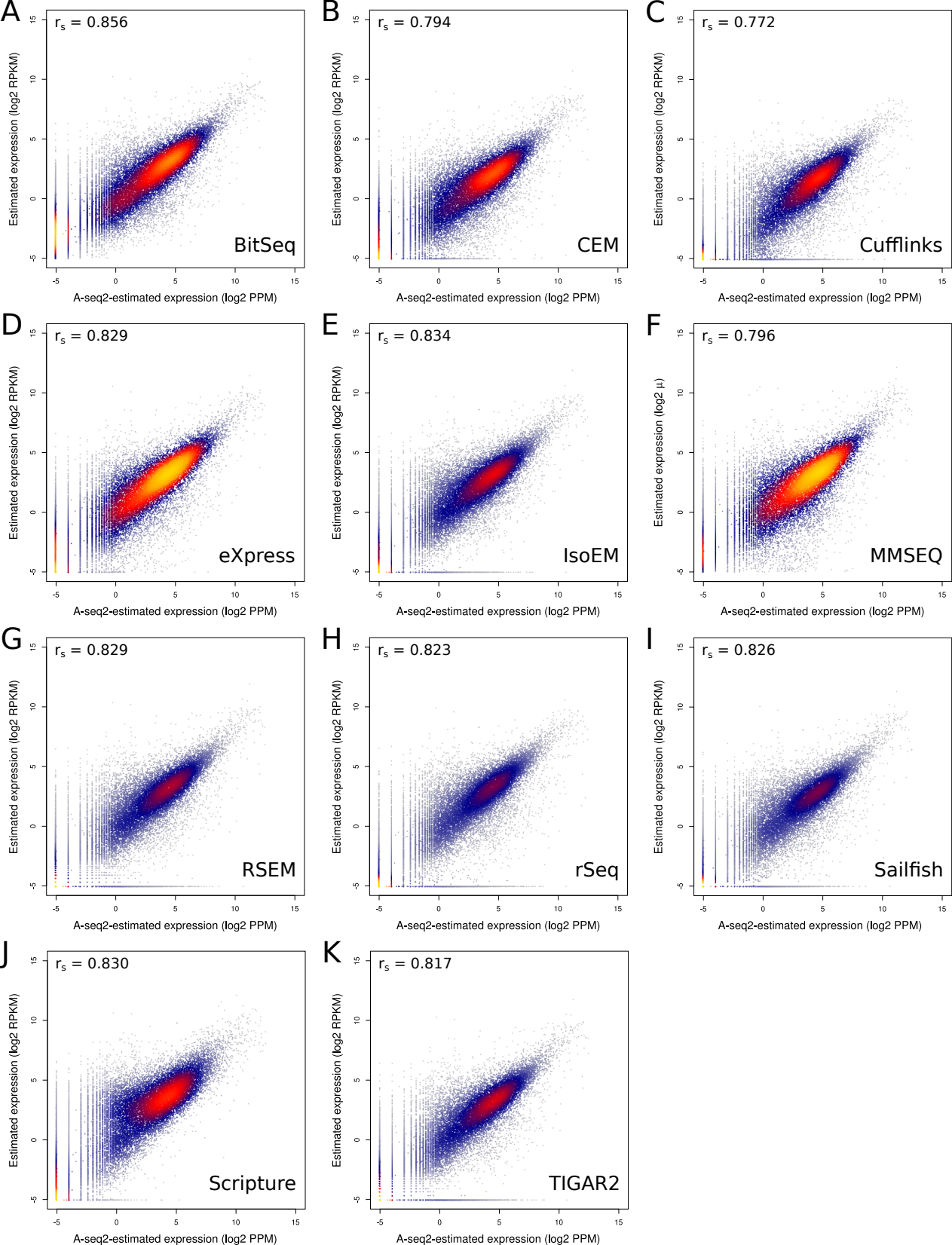

Supplement: Additional file 16: Figure S15. — Accuracy of 3′ end processing site abundance estimates inferred from Jurkat sequencing data. Transcript abundances inferred by the surveyed methods from RNA-seq libraries prepared from human Jurkat cells (replicate 1) were aggregated by 3′ end processing sites and plotted against the corresponding estimates obtained by the analysis of A-seq-2 sequencing data. Heat map colors represent data point densities and Spearman correlation coefficients (rs) are indicated. (A) BitSeq. (B) CEM. (C) Cufflinks. (D) eXpress. (E) IsoEM. (F) MMSEQ. (G) RSEM. (H) rSeq. (I) Sailfish. (J) Scripture. (K) TIGAR2. [file 13059_2015_702_MOESM16_ESM.pdf]

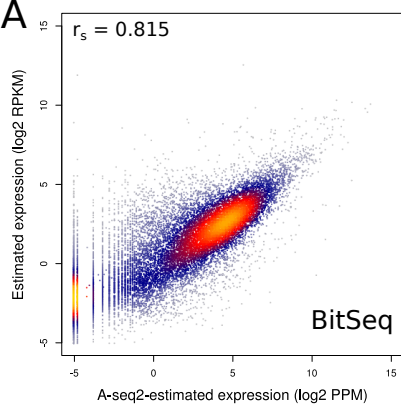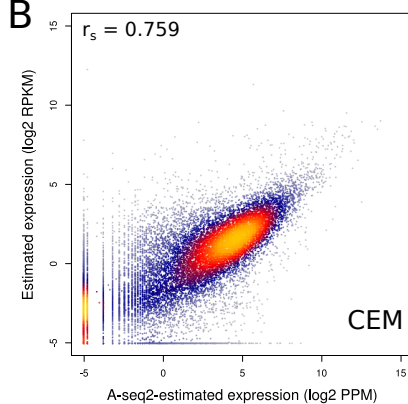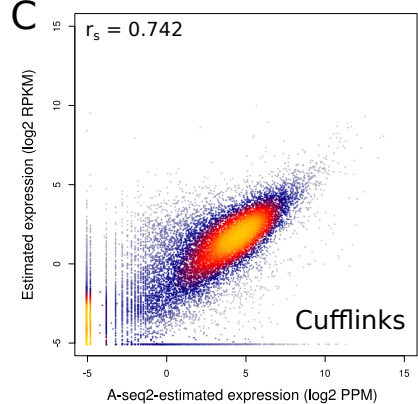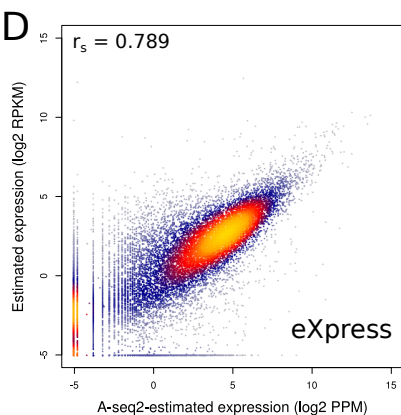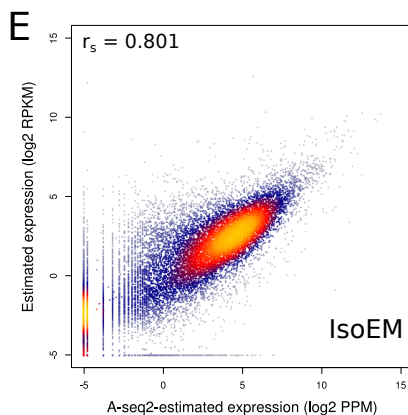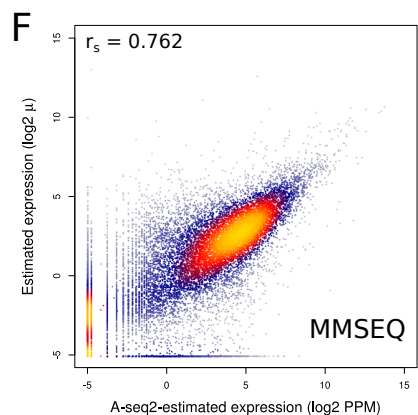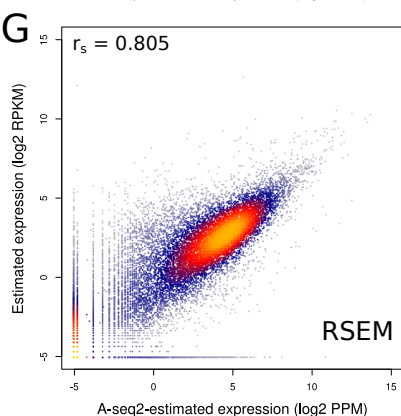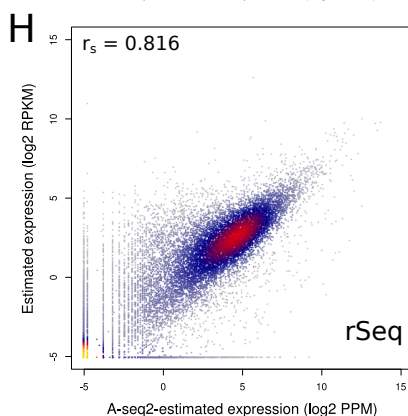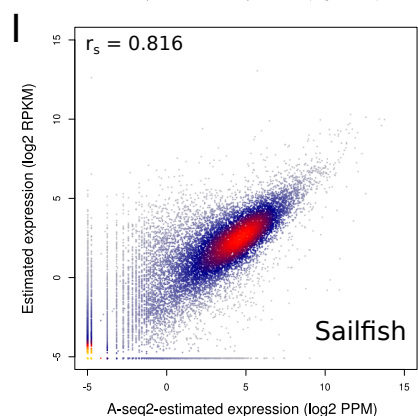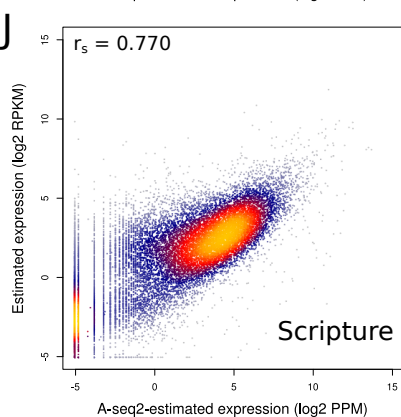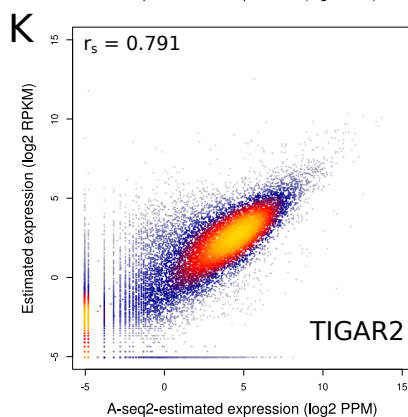

Supplement: Additional file 17: Figure S16. — Accuracy of 3′ end processing site abundance estimates inferred from NIH/3T3 sequencing data. As in Additional file 16: Fig. S15, but data were from murine NIH/3T3 cells. [file 13059_2015_702_MOESM17_ESM.pdf]
