# Supplementary material for: Comparative assessment of methods for the computational inference of transcript isoform abundance from RNA-seq data
Source: Genome Biol. 2015 Jul 23;16(1):150. doi: 10.1186/s13059-015-0702-5 (PMC4511015; doi:10.1186/s13059-015-0702-5)
Supplement: Additional file 4: Figure S4. — Comparison of different metrics for quantifying the accuracy of isoform abundance estimates. The accuracy of expression level estimates with respect to the ground truth was assessed by the Spearman and Pearson correlation coefficients, as well as the root mean square error (RMSE). The values obtained for expressed transcripts (A) and expressed genes (B) are plotted. Color intensities have been computed per column by scaling raw values such that the best value (high for correlation coefficients, low for RMSE) corresponds to the most intense and the worst to the least intense color. [file 13059_2015_702_MOESM4_ESM.pdf]

A

	Spearman					Pearson					RMSE				
	1 million reads	3 million reads	10 million reads	30 million reads	100 million reads	1 million reads	3 million reads	10 million reads	30 million reads	100 million reads	1 million reads	3 million reads	10 million reads	30 million reads	100 million reads
BitSeq	0.89	0.94	0.97	0.97	0.97	0.89	0.94	0.96	0.97	0.96	1.56	1.23	1.13	1.22	1.39
CEM	0.89	0.92	0.94	0.94	0.94	0.85	0.88	0.91	0.92	0.92	3.09	2.41	2.00	1.86	1.81
Cufflinks	0.76	0.77	0.77	0.77	0.77	0.75	0.76	0.76	0.76	0.76	4.09	3.60	3.33	3.23	3.21
eXpress	0.90	0.93	0.95	0.96	0.97	0.87	0.90	0.93	0.94	0.95	3.03	2.41	1.94	1.69	1.56
IsoEM	0.90	0.94	0.96	0.96	0.97	0.86	0.90	0.93	0.94	0.94	3.15	2.36	1.86	1.63	1.56
MMSEQ	0.90	0.92	0.93	0.93	0.93	0.85	0.89	0.90	0.90	0.90	3.75	2.91	2.31	2.03	1.87
RSEM	0.90	0.93	0.95	0.96	0.96	0.86	0.90	0.92	0.93	0.94	3.19	2.42	1.92	1.69	1.63
rSeq	0.87	0.90	0.92	0.92	0.92	0.83	0.87	0.89	0.90	0.90	3.52	2.80	2.37	2.18	2.12
Sailfish	0.89	0.93	0.95	0.96	0.96	0.86	0.89	0.92	0.93	0.94	3.22	2.50	1.99	1.79	1.67
Scripture	0.75	0.76	0.77	0.77	0.77	0.71	0.73	0.74	0.75	0.75	3.03	2.64	2.44	2.37	2.36
TIGAR2	0.90	0.93	0.95	0.96	0.96	0.86	0.90	0.92	0.94	0.94	3.27	2.50	1.99	1.74	1.65

B

	Spearman					Pearson					RMSE				
	1 million reads	3 million reads	10 million reads	30 million reads	100 million reads	1 million reads	3 million reads	10 million reads	30 million reads	100 million reads	1 million reads	3 million reads	10 million reads	30 million reads	100 million reads
BitSeq	0.90	0.95	0.98	0.99	0.99	0.86	0.93	0.97	0.98	0.98	1.92	1.36	0.94	0.81	0.86
CEM	0.93	0.95	0.95	0.95	0.96	0.87	0.90	0.92	0.93	0.93	2.10	1.63	1.37	1.31	1.29
Cufflinks	0.94	0.95	0.96	0.96	0.96	0.89	0.91	0.93	0.93	0.93	2.46	1.93	1.62	1.53	1.53
eXpress	0.96	0.97	0.98	0.99	0.99	0.91	0.94	0.96	0.97	0.97	2.29	1.71	1.31	1.13	1.03
IsoEM	0.96	0.98	0.98	0.99	0.99	0.91	0.94	0.96	0.97	0.97	2.18	1.58	1.16	1.02	0.97
MMSEQ	0.95	0.97	0.97	0.97	0.97	0.89	0.93	0.95	0.95	0.95	1.83	1.44	1.19	1.13	1.13
RSEM	0.96	0.98	0.98	0.99	0.99	0.91	0.94	0.96	0.97	0.97	2.18	1.58	1.16	1.02	0.98
rSeq	0.93	0.94	0.95	0.95	0.95	0.88	0.90	0.92	0.93	0.93	2.63	2.08	1.78	1.68	1.65
Sailfish	0.96	0.98	0.98	0.99	0.99	0.91	0.94	0.96	0.97	0.97	2.23	1.62	1.22	1.08	1.02
Scripture	0.89	0.90	0.91	0.91	0.91	0.86	0.87	0.88	0.89	0.89	2.80	2.31	2.01	1.91	1.88
TIGAR2	0.96	0.98	0.98	0.99	0.99	0.91	0.94	0.96	0.97	0.97	2.21	1.61	1.22	1.09	1.03
Count: 'Transcript'	0.87	0.88	0.89	0.89	0.89	0.86	0.88	0.88	0.89	0.89	2.95	2.69	2.57	2.54	2.53
Count: 'Union exon'	0.84	0.85	0.86	0.86	0.86	0.82	0.84	0.85	0.85	0.85	3.03	2.76	2.64	2.62	2.61
