# Supplementary material for: Comparative assessment of methods for the computational inference of transcript isoform abundance from RNA-seq data
Source: Genome Biol. 2015 Jul 23;16(1):150. doi: 10.1186/s13059-015-0702-5 (PMC4511015; doi:10.1186/s13059-015-0702-5)
Supplement: Additional file 8: Figure S8. — Effect of ‘native’ short-read aligners. For methods strongly recommending the use of a specific short-read aligner (CEM, Cufflinks, MMSEQ, Scripture) or using such an aligner internally by default (RSEM), expression levels inferred based on alignments obtained with the respective aligners were compared to the estimates produced following our own processing and alignment pipeline. Accuracies were calculated across different read depths as in Fig. 2, either for expressed transcripts (A) or genes (B), or for all transcripts (C) or genes (D). [file 13059_2015_702_MOESM8_ESM.pdf]

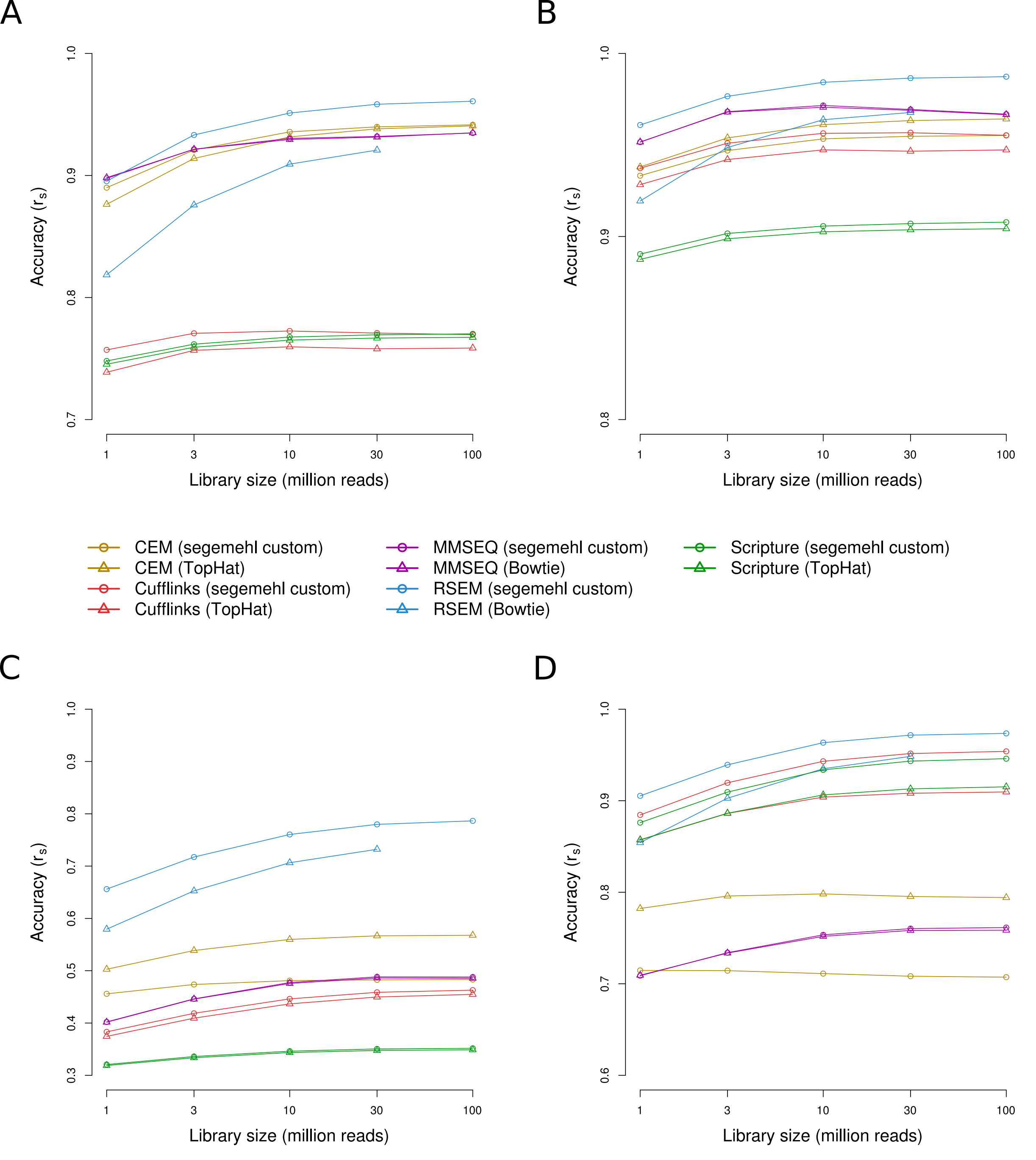
A
B
C
D
Accuracy (r_s)
Library size (million reads)
1.0
0.9
0.8
0.7
0.6
0.5
0.4
0.3
1
3
10
30
100
CEM (segemehl custom)
CEM (TopHat)
Cufflinks (segemehl custom)
Cufflinks (TopHat)
MMSEQ (segemehl custom)
MMSEQ (Bowtie)
RSEM (segemehl custom)
RSEM (Bowtie)
Scripture (segemehl custom)
Scripture (TopHat)
