# Supplementary material for: Comparative assessment of methods for the computational inference of transcript isoform abundance from RNA-seq data
Source: Genome Biol. 2015 Jul 23;16(1):150. doi: 10.1186/s13059-015-0702-5 (PMC4511015; doi:10.1186/s13059-015-0702-5)
Supplement: Additional file 9: Figure S9. — Impact of bias correction settings on simulated data. For methods where an optional sequencing/positional bias correction setting is implemented, we have compared estimation accuracies obtained when executing the programs with the respective options set or unset. Accuracies were calculated for 30 million reads as in Fig. 2, either for transcripts (A) or genes (B). Default settings (that were also used throughout this study if not indicated otherwise) are indicated in parentheses after the method name (circle: bias correction off, triangle: bias correction on). Note that Cufflinks also has a bias correction option (--frag-bias-correct; default: off). However, in our hands the program crashed when this option was specified. [file 13059_2015_702_MOESM9_ESM.pdf]

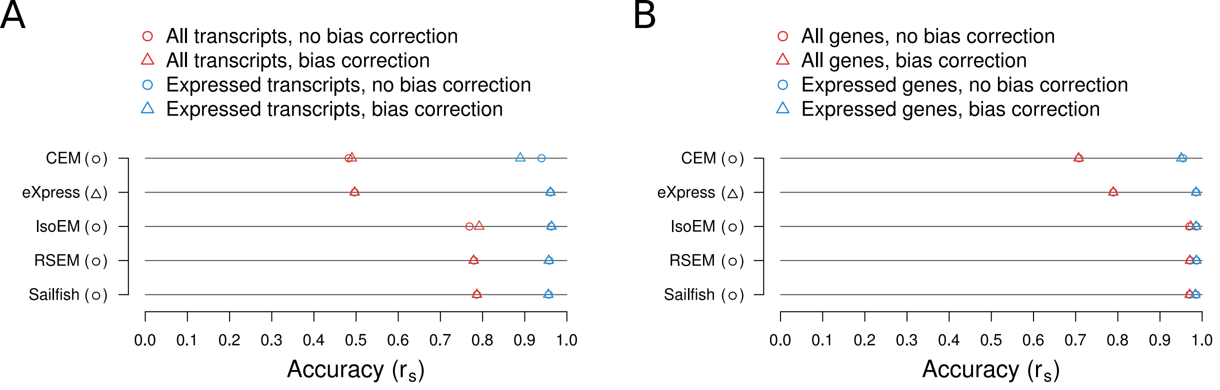
A
All transcripts, no bias correction
All transcripts, bias correction
Expressed transcripts, no bias correction
Expressed transcripts, bias correction
CEM (○)
eXpress (△)
IsoEM (○)
RSEM (○)
Sailfish (○)
0.0
0.1
0.2
0.3
0.4
0.5
0.6
0.7
0.8
0.9
1.0
Accuracy (r_s)
B
All genes, no bias correction
All genes, bias correction
Expressed genes, no bias correction
Expressed genes, bias correction
CEM (○)
eXpress (△)
IsoEM (○)
RSEM (○)
Sailfish (○)
0.0
0.1
0.2
0.3
0.4
0.5
0.6
0.7
0.8
0.9
1.0
Accuracy (r_s)
