# Supplementary material for: Comparative assessment of methods for the computational inference of transcript isoform abundance from RNA-seq data
Source: Genome Biol. 2015 Jul 23;16(1):150. doi: 10.1186/s13059-015-0702-5 (PMC4511015; doi:10.1186/s13059-015-0702-5)
Supplement: Additional file 18: Figure S17. — Impact of bias correction settings on abundance estimates from experimental data. As in Additional file 9: Fig. S9, but expression estimates were obtained for human (A and B) or mouse (C and D) cells and also include estimation accuracies on the level of 3′ end processing sites. Spearman correlation coefficients were calculated by comparison to A-seq-2 estimates (see Fig. 5) rather than the simulation ground truth. (A) Jurkat data, replicate 1. (B) Jurkat data, replicate 2. (C) NIH/3T3 data, replicate 1. (D) NIH/3T3 data, replicate 2. [file 13059_2015_702_MOESM18_ESM.pdf]

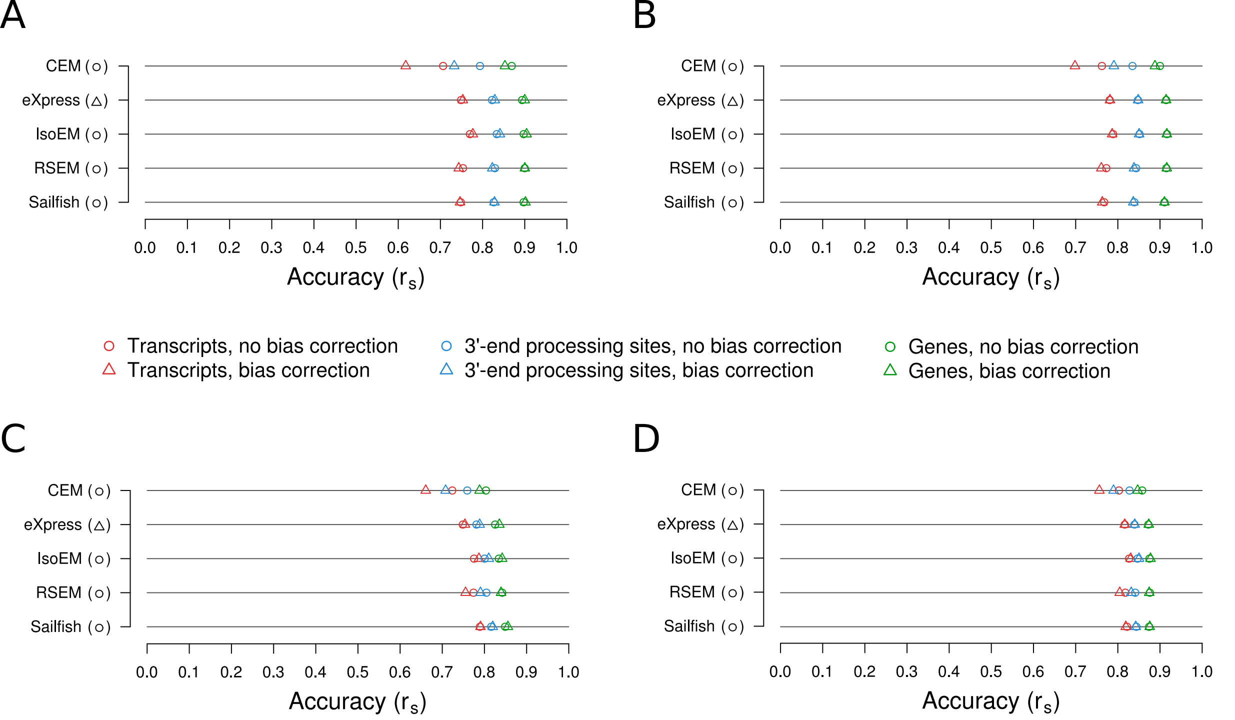
A
B
C
D
CEM (○)
eXpress (△)
IsoEM (○)
RSEM (○)
Sailfish (○)
0.0 0.1 0.2 0.3 0.4 0.5 0.6 0.7 0.8 0.9 1.0
Accuracy (r_s)
○ Transcripts, no bias correction
△ Transcripts, bias correction
○ 3'-end processing sites, no bias correction
△ 3'-end processing sites, bias correction
○ Genes, no bias correction
△ Genes, bias correction
